# Schottky barrier in pea-like Au@Bi_2_S_3_ nanoreactor enabling efficient photodynamic therapy of hepatocellular carcinoma

**DOI:** 10.1016/j.mtbio.2025.102001

**Published:** 2025-06-19

**Authors:** Liangsong Tao, Rongrong Gu, Junfa Yang, Jiewei Wang, Tiling Wu, Xianyue Rao, Hao Wang, Cheng Qian, Jian Liu, Sheng Ye, Tao Xu

**Affiliations:** a Inflammation and Immune Mediated Diseases Laboratory of Anhui Province, Anhui Institute of Innovative Drugs, School of Pharmaceutical Sciences, Anhui Medical University, Hefei, 230032, China; b Agricultural Photocatalysis Laboratory, School of Materials and Chemistry, Anhui Agricultural University, Hefei, 230036, China; c Department of Emergency Surgery, The Second Affiliated Hospital of Anhui Medical University, Hefei, 230601, China; d Department of Radiation Oncology, The First Affiliated Hospital of Anhui Medical University, Hefei, 230022, China; e Center for Scientific Research, Anhui Medical University, Hefei, 230000, China; f College of Chemistry and Chemical Engineering, Inner Mongolia University, Hohhot, 010021, China

**Keywords:** Nanoreactor, Schottky barrier, Reactive oxygen species, Hepatocellular carcinoma, Hippo/Yap signaling pathway

## Abstract

Hepatocellular carcinoma (HCC), a leading cause of tumor-related mortality globally, demands innovative therapeutic strategies to overcome limitations of conventional treatments. Photodynamic therapy (PDT), reliant on reactive oxygen species (ROS) generation, has emerged as a promising therapeutic strategy for neoplastic diseases. But it is constrained by inefficient charge separation in traditional photosensitizers. Here, we engineered a pea-like Au@Bi_2_S_3_ nanoreactors by anchoring gold nanoparticles onto Bi_2_S_3_ surfaces to establish a Schottky junction with interfacial Au-S covalent bonds, which suppresses electron-hole recombination and amplifies ROS production. Under light irradiation, Au@Bi_2_S_3_ exhibited remarkable inhibitory efficacy against HepG-2 cells, producing twice the ROS yield of Bi_2_S_3_. Transcriptomic analysis via RNA sequencing identified activation of the Hippo/Yap signaling pathway, which orchestrated endoplasmic reticulum stress and autophagic flux in malignant cells, ultimately driving apoptotic elimination of HepG-2 populations. These findings delineate a mechanistic paradigm wherein Schottky junction engineering potentiates ROS-mediated cytotoxicity, thereby advancing precise photodynamic interventions for HCC management.

## Graphical abstract

**Figure:**
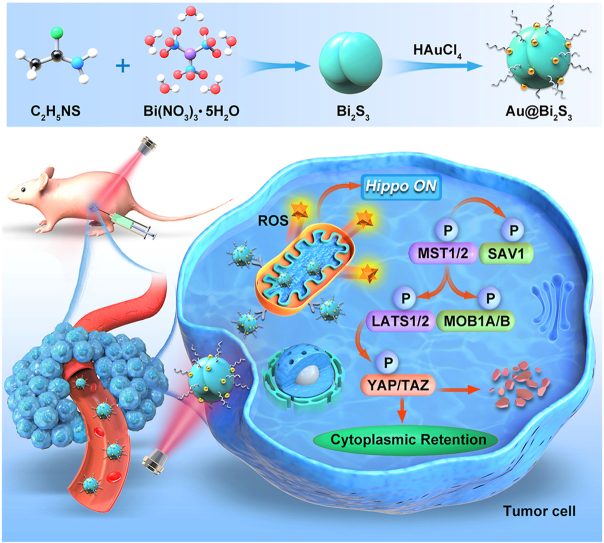

## 1 Introduction

Tumor treatment has evolved from traditional approaches such as surgical intervention and chemotherapy to advanced modalities including immunotherapy and targeted therapies, which aim to enhance therapeutic efficacy while minimizing adverse effects [1,2]. Despite these advancements, persistent challenges such as drug resistance and incomplete tumor eradication underscore the need for innovative strategies, particularly those leveraging nanotechnology. Hepatocellular carcinoma (HCC), the sixth most prevalent malignancy globally, ranks as the fourth leading cause of tumor-related mortality [3]. At present, it is widely acknowledged that the combination of genetic and environmental factors contributes to the development of this malignant tumor [4]. Liver transplantation and surgical resection remain the primary methods for the treatment of HCC [5], chemotherapy and radiotherapy are also effective for advanced HCC [6,7]. However, liver transplantation is constrained by challenges in organ acquisition and compatibility, while conventional surgical resection for HCC is associated with a high recurrence rate and poor prognosis [8]. Therefore, it is of great significance to explore novel therapeutic strategies aimed at reducing the severity and mortality of HCC.

Phototherapy, a light-based therapeutic modality, has found diverse applications across medical disciplines including dermatology, oncology, and ophthalmology [9]. This modality encompasses photodynamic therapy (PDT), photothermal therapy (PTT), laser therapy, and ultraviolet (UV) therapy, among others [10,11]. Notably, PDT represents a minimally invasive and spatially precise treatment modality [[12], [13], [14], [15]], offering distinct advantages over conventional invasive techniques [[16], [17], [18], [19]]. In PDT, localized activation of photosensitizers via light irradiation induces oxygen-dependent generation of reactive oxygen species (ROS), culminating in tumor cell lysis, vascular disruption, and subsequent tumor ablation [[20], [21], [22], [23], [24], [25], [26], [27], [28]].

Semiconductors, distinguished by their tunable electronic properties [29], play a pivotal role in modern technology. Their characteristic bandgap structure enables controlled charge carrier dynamics under external stimuli such as doping or photoexcitation [[30], [31], [32], [33]]. Bismuth sulfide (Bi_2_S_3_), a narrow bandgap semiconductor, demonstrates exceptional near-infrared (NIR) light-harvesting capabilities within the biological transparency window (700–1700 nm). This material is further characterized by robust mechanical stability, remarkable acid/alkali corrosion resistance, and favorable biocompatibility. These integrated physicochemical properties synergistically position Bi_2_S_3_ as an ideal candidate for multifunctional nanomedicine platforms, particularly in emerging theranostic strategies that unify diagnostic and therapeutic modalities [[34], [35], [36]]. Meanwhile, the precious metal nanomaterial gold (Au) is often used as a photocatalyst due to its unique optical properties and surface chemistry [[37], [38], [39], [40]]. Bi_2_S_3_, as a semiconductor with narrow band gap energy, can generate electron-hole pairs through photoexcitation. But the narrow band gap is prone to electron and hole recombination, resulting in reduced photocatalytic activity [41,42]. To address this limitation, Au nanoparticles were synthesized in situ on Bi_2_S_3_ nanoreactors surfaces to establish Schottky junctions. By controlling electron transfer at the interface, electrons accumulate on the surface of Au and inhibit the electron-hole pair recombination. Schottky heterojunction catalysts have been extensively investigated for applications spanning photovoltaics, optoelectronic sensing, energy conversion systems, and electronic device engineering [36,43,44]. The Mott-Schottky effect has begun to attract attention in the biomedical field in recent years, which originates from the electron interaction between metals and semiconductors and can regulate the charge transfer and catalytic activity at the interface. However, there are currently very few related reports. Huang et al. prepared a bismuth-based nanosonosensitizer (Bi-HJ) for combined tumor therapy. A Schottky barrier and oxygen vacancies were simultaneously constructed in Bi-HJ, which can promote the separation of electron-hole pairs triggered by ultrasound and realize sonodynamic therapy [45]. In terms of antibacterial applications, the Schottky barrier designed in the nanozyme system has been proven to accelerate the generation of ROS by optimizing the separation of electron-hole pairs to achieve the inactivation of microorganisms [46]. These studies highlight the versatility of the Mott-Schottky system in addressing biomedical challenges.

Herein, our team designed and constructed an effective photosensitizer: an Au@Bi_2_S_3_ composite nanomaterial, which enables efficient PDT of HCC by leveraging the Schottky barrier effect. This study demonstrated that under the illumination condition, Au@Bi_2_S_3_ nanoreactors produced a large number of ROS, which activated the Hippo/Yap signaling pathway, induced endoplasmic reticulum (ER) stress and autophagy of tumor cells, thus leading to the apoptosis of HepG-2 cells. When XMU-MP-1, an inhibitor of Hippo/Yap signaling pathway, was used, the apoptosis of HepG-2 cells was reduced.

## 2 Materials and methods

### 2.1 Synthesis of Bi_2_S_3_

50 mg of bismuth nitrate pentahydrate was dissolved in 50 mL of pure water and stirred for 2 h. Then, 27 mg of thioacetamide was added and stirred for another 12 h. After that, it was transferred to a 100 mL polytetrafluoroethylene reactor and subjected to a hydrothermal reaction at 160 °C for 8 h. After the reaction is completed, cool, wash with water and ethanol multiple times, centrifuge, and finally dry the product at 60 °C to obtain Bi_2_S_3_ nanoreactors.

### 2.2 Synthesis of Au@Bi_2_S_3_

20 mg of Bi_2_S_3_ was dispersed in a mixed solution of 27 ml of ethanol and 9 ml of water. Subsequently, 6 mL of aqueous solution containing 0.98 mg of chloroauric acid and 40 mg of AOT cellulose were added dropwise to the mixed solution, stirred for 10 min, 0.2 g of L-ascorbic acid was added, stirred for another 12 h, and then subjected to a 12 h hydrothermal reaction at a temperature of 80 °C. Wash the black product repeatedly with water and ethanol, centrifuge and separate, and dry at room temperature. Then it was heated at 80 °C in a tube furnace for 3 h (Anhui Kemi Machinery Technology Co., ltd. BFC-1200).

### 2.3 Synthesis of Au@Bi_2_S_3_-PEG

50 mg of Au@Bi_2_S_3_ was added to 3 mL of EtOH and sonicated to obtain solution A. 100 mg of LA-PEG (MW: 5k) was dissolved in 1 mL of H_2_O to obtain solution B. Solution B was slowly added dropwise to solution An under continuous stirring, followed by further stirring for 12 h. After centrifugation, LA-PEG-modified Au@Bi_2_S_3_ was collected.

### 2.4 Cell culture

HCC cell lines (HepG-2) and L-02 cells were purchased from ATCC (Manassas, VA, USA) and were cultured in Dulbecco's Modified Eagle Medium (DMEM) containing 10 % fetal bovine serum (Gibco, Waltham, MA, USA) and 100 U/mL penicillin-streptomycin (Gibco). The culture environment was maintained at 5 % CO_2_ and 37 °C in humidified air. The culture medium was changed three times per week.

### 2.5 Animals and tumor model

Female BALB/c nude mice at 6–8 weeks of age were obtained from GemPharmatech (Nanjing, China). All animals were cared in accordance with the guidelines in the Guide to the Care and Use of Experimental Animals, and all procedures were approved by the Animal Care and Use Committee of Anhui Medical University. 1 × 10^6^ cells (100 μL) were injected into the right side of BALB/c nude mice to form a xenograft tumor model. All animal testing procedures have been approved by the ethical guidelines and reviewed and implemented in accordance with the standards of the Experimental Animal Ethics Committee of the First Affiliated Hospital of USTC (2023-N (A) −78).

### 2.6 ROS detection in vitro

The ROS level in cells was determined by a ROS Assay Kit (Beyotime, China) containing the fluorescence reporter probe 2′,7′-dichlorodihydrofluorescein diacetate (DCFH-DA). Cells were incubated with different agents for 24 h, and then fresh medium without serum-containing DCFH-DA (10 μM, Beyotime) was added and incubated for a further 30 min. Then the cells were washed three times to remove DCFH-DA. Lastly, the green fluorescence of 2′,7′-dichlorofluorescein (DCF) was observed by an inverted microscope. For detection with Mito-Tracker Deep Red FM, a working solution (100 nM) was prepared and incubated with the cells for 30 min at 37 °C. After the incubation, remove the working solution and add fresh cell culture medium pre-incubated at 37 °C. The stained cells were then observed or detected using a fluorescence microscope, laser confocal microscope, or fluorescence plate reader.

### 2.7 Biosafety assessment

Biosafety assessment of the compounds was performed as previously described. In brief, to evaluate the biosafety of Bi_2_S_3_ or Au@Bi_2_S_3_, blood samples were collected, and corresponding serum biochemistry tests (including ALT and AST) were performed after 7 days of various treatments.

### 2.8 Cell viability detection

HepG-2 and L-02 cells were used to investigate the cytotoxicity of Bi_2_S_3_ or Au@Bi_2_S_3_ by Cell Counting Kit-8 (CCK-8, Yeasen, Shanghai, China) according to the manufacturer's instructions. Briefly, cells were seeded into 96-well plates at 5 × 10^3^ cells/well and then added to the medium containing various concentrations of Bi_2_S_3_ or Au@Bi_2_S_3_. Following incubation for 24 h, cell viability was tested by adding 10 μL of CCK-8 to each well and incubating for a further 90 min. Then, the absorbance (Abs) at 450 nm was measured to calculate cell viability. To assess the impact on cell proliferation, the HepG-2 and L-02 cells were irradiated under light (λ > 420 nm, 0.4 W/cm^2^) in 30 min after incubation with Bi_2_S_3_ or Au@Bi_2_S_3_ for 24 h. The cell viability was obtained similarly after further incubation for 24 h.

### 2.9 Colony formation assay

A colony formation assay was performed to evaluate cell proliferation in the seven groups. Approximately 1000–1500 cells were placed into six-well plates after being given corresponding treatments and cultured for 12 days. Afterward, cells were stained with crystal violet, and then the numbers of colonies were calculated and analyzed.

### 2.10 Ethics approval statement

All animal testing procedures have been approved by the ethical guidelines and reviewed and implemented in accordance with the standards of the Experimental Animal Ethics Committee of the First Affiliated Hospital of USTC (2023-N (A) −78).

### 2.11 Statistical analysis

Data were indicated as the mean ± SD. The experimental data and differences were analyzed using GraphPad Prism 9.3 software (GraphPad, San Diego, CA) with a one‐way analysis of variance (ANOVA) with Tukey's multiple comparisons test. Significant differences among groups were defined as *∗p< 0.05, ∗∗p< 0.01 and ∗∗∗p< 0.001, ∗∗∗∗p< 0.0001*, respectively. *p* < 0.05 was considered to have a significant difference (95 % confidence level).

## 3 Results and discussion

### 3.1 Synthesis and characterization of Au@Bi_2_S_3_ nanoreactors

**Fig. 1:**
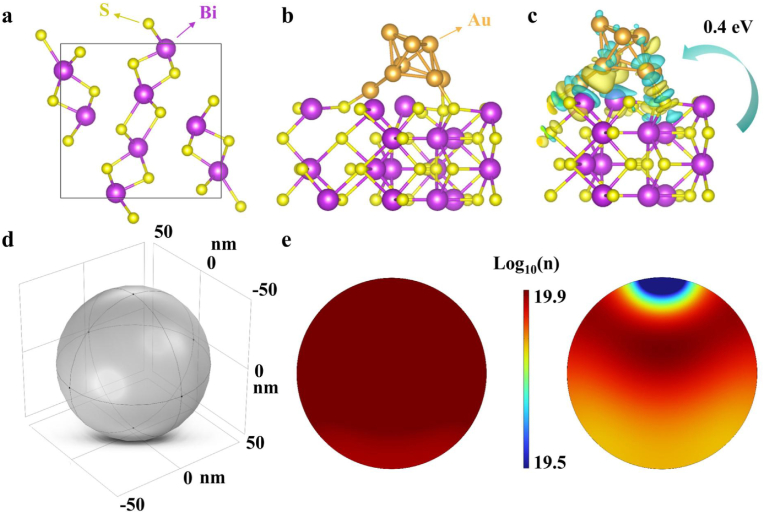
a): Cell structure of Bi_2_S_3_; b): The molecular structure model of Au@Bi_2_S_3_; c): Au@Bi_2_S_3_ electron density distribution; d): Simplified 3D particle model of Bi_2_S_3_; e): Two-dimensional cross section of electron density of Bi_2_S_3_ and Au@Bi_2_S_3_ under light.

**Fig. 2:**
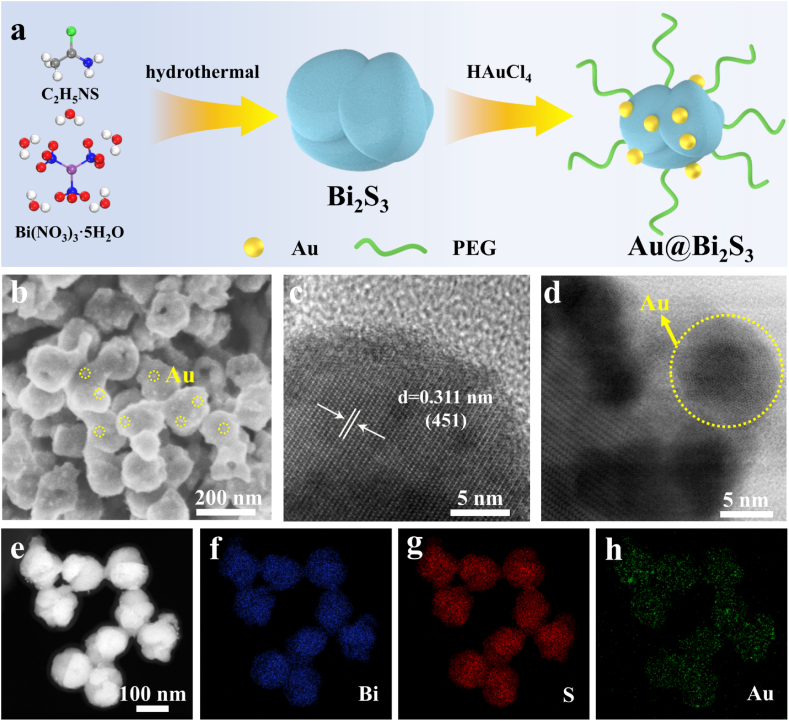
a): The process of Au@Bi_2_S_3_ preparation; b): The SEM of Au@Bi_2_S_3_ nanoreactors; c): The HRTEM of Au@Bi_2_S_3_ nanoreactors; d): Spherical aberration corrected transmission electron microscopy of Au@Bi_2_S_3_ nanoreactors; e), f), g) and h): The corresponding SEM mapping of Au@Bi_2_S_3_ nanoreactors.

The molecular and electronic configurations of Bi_2_S_3_ and Au@Bi_2_S_3_ heterojunctions were systematically investigated through density functional theory (DFT) calculations and COMSOL multiphysics simulations. Fig. 1a depicted the cell structure of Bi_2_S_3_. The molecular model of the Au@Bi_2_S_3_ catalyst demonstrated covalent bonding between Au atoms and S atoms within the Bi_2_S_3_ framework, forming a stable heterojunction interface (Fig. 1b). In the 3D model diagram of Fig. 1c, the yellow region represented the decrease of electron density, while the cyan region represented the increase of electron density. Electrons were primarily distributed between S-Au bonds and are closer to the Au atom, creating an electron-deficient region near the S atom. This indicated that electrons were transferred from the S atom to the Au atom, with an electron transfer valued at 0.4 eV. The addition of Au atoms altered the electron distribution throughout the catalyst, and this change in electronic structure enhanced its catalytic performance. Moreover, the photogenerated electron densities of Bi_2_S_3_ and Au@Bi_2_S_3_ were simulated by COMSOL simulation. After the introduction of Au, the electron concentration of Bi_2_S_3_ at the contact site decreased, indicating that electrons are transferred from Bi_2_S_3_ to Au (Fig. 1d and e). The Bi_2_S_3_ nanoreactors and Au@Bi_2_S_3_ nanoreactors were synthesized via a hydrothermal method using bismuth nitrate as the Bi precursor and thioacetamide as the sulfur source (Fig. 2a). Then Au nanoparticles were grown in situ on the surface of pea-like Bi_2_S_3_ using chlorauric acid as the Au source. Surface modification of Au@Bi_2_S_3_ with hydrophilic polyethylene glycol (PEG) significantly improved biocompatibility. X-ray diffraction (XRD) showed that all diffraction peaks correspond well to Bi_2_S_3_ (PDF#17–0320). Due to the low content and high dispersion, the diffraction peak of Au was not observed. Additionally, there was no obvious difference between Bi_2_S_3_ and Au@Bi_2_S_3_ in Raman peaks (Figs. S1 and S2). Scanning electron microscope (SEM) images showed that Bi_2_S_3_ and Au@Bi_2_S_3_ nanoreactors had diameters ranging from about 110 nm to 180 nm (Fig. 2b–S3, S5), and Au nanoparticles were uniformly distributed onto pea-like Bi_2_S_3_. High resolution transmission electron microscope (HRTEM) results revealed that the lattice spacing is 0.311 nm, corresponding to the (451) crystal plane of Bi_2_S_3_ (Fig. 2c). Fig. 2d depicted a spherical aberration corrected transmission electron microscopy (AC-TEM) image of Au@Bi_2_S_3_ nanoreactors, which showed that the diameter of Au is approximately 10 nm (Figs. S4, S6, S8). The element distribution diagram of Au@Bi_2_S_3_ showed that Bi, S and Au elements were evenly distributed (Fig. 2e–h, S7). As shown in Fig. S9, the peak at 1619 cm^−1^ corresponded to the N-H stretching vibration of the amino group, while the signals at 1275 cm^−1^ and 1238 cm^−1^ corresponded to C-O and C-N bonds, respectively. The Fourier transform infrared (FT-IR) spectral data confirmed the successful loading of LA-PEG onto the surface of Au@Bi_2_S_3_.

**Fig. 3:**
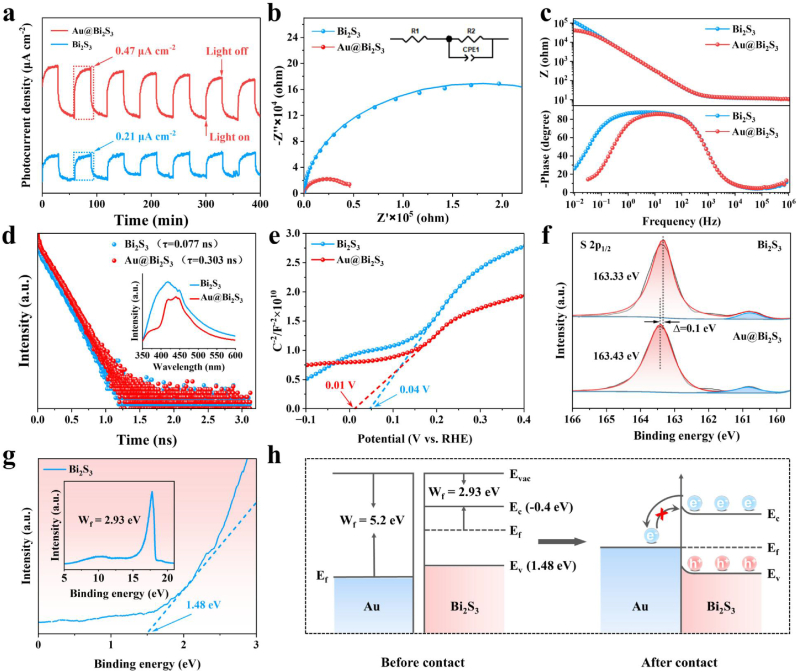
a): The Current against time for on−off cycles for Bi_2_S_3_ and Au@Bi_2_S_3_ with xenon lamp source; b): Electrochemical impedance spectroscopy of Bi_2_S_3_ and Au@Bi_2_S_3_; c) Bode phase plots of Bi_2_S_3_ and Au@Bi_2_S_3_; d): The TRPL spectra and the PL spectra of Bi_2_S_3_ and Au@Bi_2_S_3_ nanoreactors (insets); e): Mott-Schottky curves of Bi_2_S_3_ and Au@Bi_2_S_3_; f): XPS high-resolution of S 2p for Bi_2_S_3_ and Au@Bi_2_S_3_, respectively; g): UPS spectra of Bi_2_S_3_; h): Electron transfer at the interface of Au@Bi_2_S_3_.

The transient photocurrent test showed that the photocurrent density of Au@Bi_2_S_3_ was twice that of Bi_2_S_3_, demonstrating that the introduction of Au enhanced the efficient separation of photogenerated electron-hole pairs, which was likely attributed to the transfer of electrons from Bi_2_S_3_ to the Au surface (Fig. 3a). The Nyquist plot showed that Au@Bi_2_S_3_ had lower charge transfer resistance compared to Bi_2_S_3_ (Fig. 3b). Furthermore, the Bode plots showed that the resistance values of both sides decrease at different frequencies, and the phase Angle of Au@Bi_2_S_3_ moved towards high frequency, exhibiting a smaller peak phase Angle. This indicated that the combination of Au and Bi_2_S_3_ could promote the rapid transfer of electric charge (Fig. 3c). Time-resolved photoluminescence spectroscopy (TRPL) could be used to calculate the carrier lifetime, and the photoluminescence (PL) measures the photons emitted by the radiative recombination of photogenerated electrons and holes. As shown in Fig. 3d, the mean fluorescence lifetimes of Au@Bi_2_S_3_ and Bi_2_S_3_ are 0.303 ns and 0.077 ns, respectively. In the illustration, the photoluminescence intensity of Au@Bi_2_S_3_ is lower than that of Bi_2_S_3_, indicating that the photogenerated electron-hole pair recombination rate of Au@Bi_2_S_3_ is lower, which may be due to the Schottky barrier formed between Au and Bi_2_S_3_. The Mott-Schottky (MS) experiment is carried out to measure flatband potentials for Bi_2_S_3_ and Au@Bi_2_S_3_, recorded at a constant frequency of 1000 Hz (Fig. 3e–S12). The flat-band potential was determined by extrapolating the tangent of the Mott-Schottky plot to its intersection with the horizontal axis. The flat-band potentials of Bi_2_S_3_ and Au@Bi_2_S_3_ are 0.04 V and 0.01 V, respectively. To elucidate the electron transfer of Au@Bi_2_S_3_, the Bi (Fig. S10) and S (Fig. 3f) elements in Bi_2_S_3_ and Au@Bi_2_S_3_ were characterized by X-ray photoelectron spectroscopy (XPS). The peaks of Bi_2_S_3_ at 163.37 eV and 158.07 eV belong to 4f_5/2_ and 4f_7/2_ of Bi, respectively, and the peaks of Au@Bi_2_S_3_ at 163.44 eV and 158.14 eV belong to 4f_5/2_ and 4f_7/2_ of Bi, respectively. Interestingly, the S 2p peak of Au@Bi_2_S_3_ is shifted by 0.1 eV in the direction of increased binding energy compared with Bi_2_S_3_, indicating a significant electron transfer. But the effect on the Bi 4f peak is minimal. To further reveal the interaction between Au and Bi_2_S_3_ in Mott-Schottky heterojunction, the work function (WF), valence band (VB) and conduction band (CB) of Bi_2_S_3_ are calculated using ultraviolet photoelectron spectroscopy (UPS) and UV–vis diffuse reflectivity spectra. The work function (Φ) is the minimum energy required for electrons to move from the Fermi level to the vacuum level and can control the direction of charge transport at the interface.

As shown in Fig. 3g, the work function of Bi_2_S_3_ is 2.93 eV **(inset of** Fig. 3g**)**, and the VB value is 1.48 eV. According to the band gap of Bi_2_S_3_ shown in Tauc plot is 1.88 eV, the CB value can be calculated as −0.4 eV (Fig. S11). Combined with UPS experiment, the electrons in the VB of Bi_2_S_3_ are excited to the CB under light. The formation of a Schottky barrier at the Au and Bi_2_S_3_ interface induces downward band bending in Bi_2_S_3_. Driven by the work function difference, electrons in the CB of Bi_2_S_3_ spontaneously transfer to the Au nanoparticles. Moreover, it is difficult for these electrons to return from Au to Bi_2_S_3_, thus inhibiting the electron-hole pair recombination (Fig. 3h). According to the open-circuit potential (OCP) value, the transient transfer lifetime of charge carriers was calculated (Fig. S13). The transient transfer lifetime of Au@Bi_2_S_3_ was 30 ms, which was lower than that of Bi_2_S_3_ (180 ms), indicating that the presence of the Schottky barrier enables the charge transfer in a shorter time.

### 3.2 Cytotoxicity in vitro

**Fig. 4:**
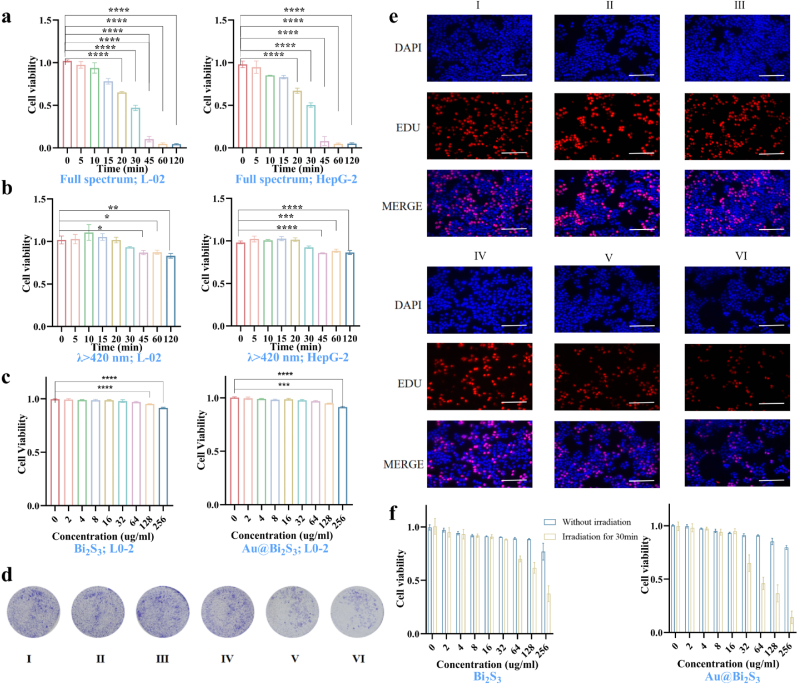
a): CCK-8 was used to evaluate phototoxicity. (0.4 W/cm^2^); b): L-02 cells were used to assess the toxicity of Bi_2_S_3_ and Au@Bi_2_S_3_ on normal liver cells across a concentration range of 0–256 μg/mL; c): HepG-2 cells were used to evaluate the impact of Bi_2_S_3_ and Au@Bi_2_S_3_ on cell viability in HCC cells across the same concentration range; d): Clone formation assay was employed to examine the effect of six different treatments on the proliferation of HepG-2 cells; e): EDU fluorescence staining was used to examine the impact of six different treatments on the proliferation of HepG-2 cells; f): CCK-8 was used to evaluate the effect of Bi_2_S_3_ and Au@Bi_2_S_3_ on cell viability of HepG-2 cells under light exposure and no light exposure within the concentration range of 0–256 μg/mL (λ > 420 nm, 30 min, and 0.4 W/cm^2^) All results of this study were derived from three independent experiments. *∗p< 0.05, ∗∗p< 0.01, ∗∗∗p< 0.001, ∗∗∗∗p< 0.0001.* Error bars represent SEM. I: Control; Ⅱ: Light for 30 min; III: Bi_2_S_3_; IV: Au@Bi_2_S_3_; V: Bi_2_S_3_ under light; VI: Au@Bi_2_S_3_ under light.

To assess the safety of light, a range of light durations (0, 30, 45, 60, 120 min) were established. Cell viability was quantified via the cell counting kit-8 (CCK-8) assay. After brief exposure to full-spectrum light, the cell viability of human normal hepatocyte L-02 cells decreased rapidly, falling below 50 % within 10 min. Considering the impact of UV radiation on cell viability, it was observed that cell viability remained above 95 % for the 30 min after the introduction of the 420 nm filter. The results indicated that full-spectrum light exposure exhibited some toxicity due to significant UV-induced cellular damage. In contrast, the addition of the 420 nm filter significantly reduced phototoxicity, causing almost no damage to the cells within the first 30 min (Fig. 4a and b). In order to determine the light intensity, HepG-2 cells and L-02 cells were irradiated with different light intensities respectively. It was found that the cell viability remained above 95 % when the maximum light intensity was 0.4 W/cm^2^ (Fig. S17a and S17b). The development of biomedical nanomaterials necessitates stringent evaluation of biocompatibility and toxicological profiles. Thus, the cytotoxicity of the Bi_2_S_3_ and Au@Bi_2_S_3_ nanoreactors was assessed on both L-02 cells and HepG-2 cells.

The CCK-8 assay was employed to detect the proliferation viability of the L-02 cells and HepG-2 cells after being incubated with Bi_2_S_3_ and Au@Bi_2_S_3_ nanoreactors at various concentrations (0, 32, 64, 128, and 256 μg/mL) for 24 h. The cell viability remained above 90 % when the concentrations of Bi_2_S_3_ and Au@Bi_2_S_3_ nanoreactors were below 128 μg/mL. The results indicated that both Bi_2_S_3_ and Au@Bi_2_S_3_ nanoreactors exhibited low cytotoxicity and good biocompatibility in vitro (Fig. 4c). In order to verify the internalization of Au@Bi_2_S_3_, the intrinsic fluorescence characteristics of Au@Bi_2_S_3_ were used to directly observe its distribution in cells, and DAPI (blue channel) was used to label the nucleus to realize multi-channel fluorescence co-localization. HepG-2 cells were incubated with Au@Bi_2_S_3_ (64 μg/mL) for 6 h. After fixing the cells, the aggregated fluorescence of Au@Bi_2_S_3_ in the cell membrane and cytoplasm was observed by laser confocal microscope, and mainly distributed around the nucleus (Fig. S29). This result showed that the nanoreactors were internalized into HepG-2 cells.

### 3.3 Antitumor activity in vitro

To preliminarily investigate the in vitro antitumor efficacy of the Au@Bi_2_S_3_ nanoreactors, we systematically assessed their impact on HCC cell proliferation. As illustrated in Fig. 4, the cell viability decreased when the Bi_2_S_3_ or Au@Bi_2_S_3_ nanoreactors were co-incubated with the cells under light exposure. Notably, in the Au@Bi_2_S_3_ group co-incubated with light, cell viability decreased more significantly, revealing a synergistic enhancement of tumor cell eradication with the introduction of Au. To optimize treatment parameters while minimizing off-target effects, subsequent experiments employed 64 μg/mL Au@Bi_2_S_3_ with 30 min light exposure (Fig. 4f). The EDU staining results showed that the proliferation rate of HepG-2 cells treated with Au@Bi_2_S_3_ and Bi_2_S_3_ under light was significantly reduced compared to dark treatments (Fig. 4e).

**Fig. 5:**
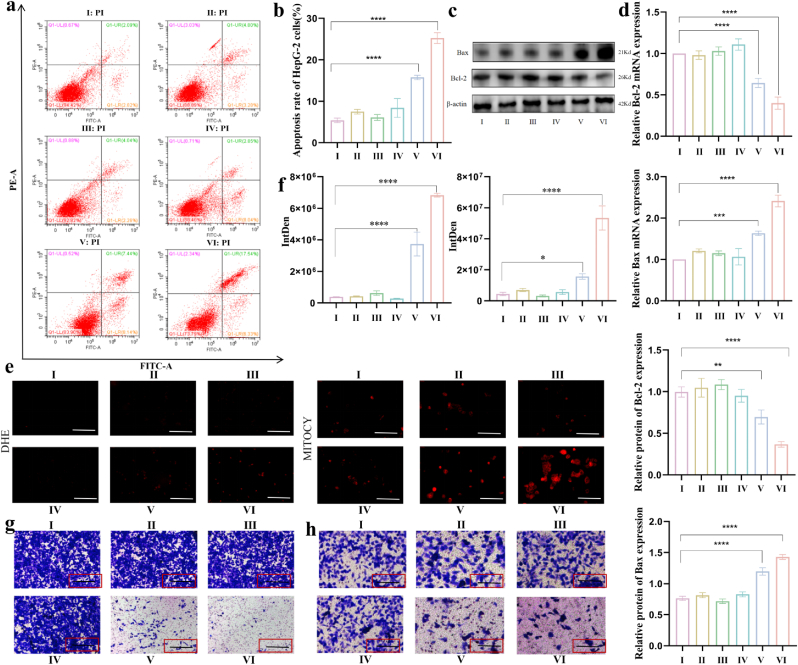
a, b):The flow cytometry was used to analyze the impact of six distinct treatments on the apoptosis of HepG-2 cells and its quantification was evaluated; c, d): Western blotting and RT-qPCR were used to detect the alterations in apoptosis-related proteins and genes of Bax and Bcl-2 in HepG-2 cells subjected to six different treatments; e, f): Dihydroethidium (DHE) fluorescent probe and Mito-Tracker Deep Red FM probe were used to examine the ROS production in HepG-2 cells; g): Transwell assay was used to evaluate the changes in the migration capacity of HepG-2 cells. h): Transwell assay was used to detect the alterations in the invasion capability of HepG-2 cells. All results of this study were derived from three independent experiments. *∗p< 0.05, ∗∗p< 0.01, ∗∗∗p< 0.001, ∗∗∗∗p< 0.0001.* Error bars represent SEM. I: Control; Ⅱ: Light for 30 min; III: Bi_2_S_3_; IV: Au@Bi_2_S_3_; V: Bi_2_S_3_ under light; VI: Au@Bi_2_S_3_ under light.

Meanwhile, the colony formation results were consistent with the EDU staining experiments, showing that treatment with Au@Bi_2_S_3_ inhibits the proliferation of HepG-2 cells under light (Fig. 4d). Interestingly, the proliferation rate of HepG-2 cells treated with Au@Bi_2_S_3_ decreased more significantly than those treated with Bi_2_S_3_ under light. The expression of the proliferation-related protein PCNA showed significant down-regulation after treatment with the two composites combined with light exposure (Fig. S18a and S18b). The corresponding detection results showed that the changes in PCNA mRNA expression were consistent with the changes in protein expression (Fig. S18c). To further substantiate their inhibitory effect on HCC in vitro, cell apoptosis and cell cycle progression were assessed using flow cytometry. The cell cycle imbalance is a primary characteristic of tumor [47]. In tumor pathology, the proportion of S-phase cells is often used as a marker to evaluate the proliferative state of a tumor [48]. The findings revealed a prominently higher apoptotic rate of HepG-2 cells treated with Au@Bi_2_S_3_ under light compared to other treatments, particularly in the early stage, demonstrating at least a twofold increase compared to other treatments (Fig. 5a and b). Flow cytometry was used to detect the number of cells in the G0/G1, S, and G2/M phases of the cell cycle. The results indicated that S-phase cells were decreased when treated with Au@Bi_2_S_3_ under light compared with the control group (Fig. S19). The results of TUNEL staining demonstrated that the apoptotic rate of HepG-2 cells treated with Au@Bi_2_S_3_ and Bi_2_S_3_ under light was significantly higher compared with the dark treatments (Fig. S20).

There is a state of hypoxia in the tumor, which may affect the curative effect. Thus, the PDT efficacy of Au@Bi_2_S_3_ will be discussed under hypoxic conditions. The results of Western blotting showed that the expression of hypoxia index protein HIF-1α was upregulation in hypoxic status (Fig. S27a). CCK-8 assay was used for PDT assessment. HepG-2 cells were treated with Au@Bi_2_S_3_ (64 μg/mL) for 6 h, followed by irradiation with a light source (0.4 W/cm^2^, 30 min). The results showed that the cell viability of the Au@Bi_2_S_3_ under light treatment group was 77 % under anoxic condition, which was significantly different from that under normoxic condition (41 %), indicating that its therapeutic effect was related to oxygen supply (Fig. S27b). However, compared with the treatment group without light under anoxic conditions, the treatment of Au@Bi_2_S_3_ with light under anoxic environment also had a certain tumor inhibition effect, showing a partial decline in the activity of HepG-2 cells. To sum up, when in hypoxia, although the nanoreactors had a certain tumor inhibition effect, it was obviously inhibited compared with the normal oxygen condition. On this surface, the nanoreactors had certain oxygen dependence.

In contrast, the apoptotic rate of HepG-2 cells treated with Au@Bi_2_S_3_ was elevated to a more pronounced extent compared to the treatment with Bi_2_S_3_ under light. Subsequently, the related apoptotic proteins were detected by Western blotting and RT-qPCR. The results displayed a significant decrease in the expression levels of Bcl-2 and a concurrent increase in the expression levels of Bax when treated with Au@Bi_2_S_3_ and Bi_2_S_3_ under light compared to the dark treatments. This suggested that the treatment involving materials combined with light markedly enhanced the apoptosis of HepG-2 cells. Of note, the increase in the apoptotic rate of HepG-2 cells treated with Au@Bi_2_S_3_ was more pronounced than the treatment with Bi_2_S_3_ under light (Fig. 5c and d). These results preliminarily indicated that Bi_2_S_3_ and Au@Bi_2_S_3_ had certain effects on the proliferation and apoptosis of HepG-2 cells in vitro when combined with phototherapy. Next, to investigated the influence of Au@Bi_2_S_3_ on the migration and invasion of HepG-2 cells, a Transwell assay was performed to determine if different treatments could enhance the inhibitory effect. The results demonstrated that the migration and invasion of HepG-2 cells treated with Au@Bi_2_S_3_ under light were most significantly inhibited compared to other treatments (Fig. 5g and h). These results indicated that the proliferation, invasion, and migration of HepG-2 cells were significantly inhibited by Au@Bi_2_S_3_ accompanied by light treatment, and the apoptotic rate was increased.

### 3.4 Biological safety evaluation in vivo

**Fig. 6:**
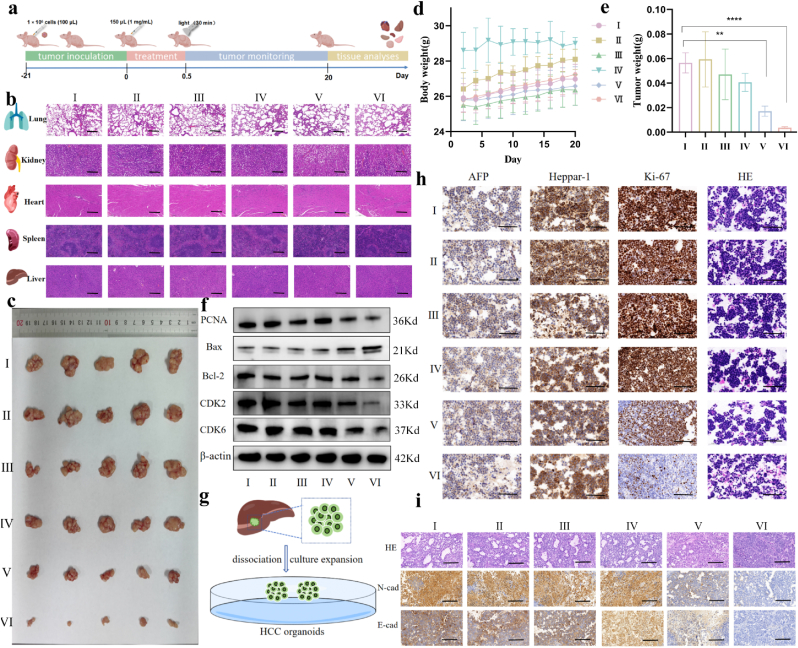
a): The flow chart was drawn for the verification of anti-HCC activity in vivo; b): H&E staining was used to evaluate the damage degree of important organs in mice, including lungs, kidneys, hearts, spleens, and livers; c, e): The subcutaneous tumors were removed and analyzed for tumor weight across different groups; d): The body weight of mice was monitored over the 20-day treatment period; f): Western blotting was used to detect the expression changes of tumor-related proteins including the protein PCNA related to proliferation, Bax and Bcl-2 related to apoptosis, and CDK2 and CDK6 related to the cell cycle; g): The flow chart of HCC organoids construction was drawn; h): H&E staining was used to evaluate the damage of HCC organoids and immunohistochemistry was used to verify the related expression indexes of HCC organoids, including AFP, Heppar-1 and Ki67; i): H&E staining was chosen to observe the mice tumor and immunohistochemistry was used to detect the changes in tumor-related indicators including tumor invasion and migration related proteins E-cad and N-cad. All results of this study were derived from three independent experiments. *∗p< 0.05, ∗∗p< 0.01, ∗∗∗p< 0.001, ∗∗∗∗p< 0.0001.* Error bars represent SEM. I: Control; Ⅱ: Light for 30 min; III: Bi_2_S_3_; IV: Au@Bi_2_S_3_; V: Bi_2_S_3_ under light; VI: Au@Bi_2_S_3_ under light.

The biosafety of drugs is important in driving research toward practical applications. The results of the above in vitro studies showed negligible toxicity of both Au@Bi_2_S_3_ and Bi_2_S_3._ Next, their biological toxicity was tested in mice. Firstly, after a 7-day treatment period, parameters related to liver and renal function, including Alanine aminotransferase (ALT) and Aspartate aminotransferase (AST), were assessed. Notably, none of these markers exhibited visible differences among the various treatment groups (Fig. S21). Subsequently, H&E staining was conducted on various organs, including the heart, lung, liver, spleen, and kidney (Fig. 6b). There was no significant difference in body weight among the six groups during the treatment, indicating no obvious side effects (Fig. 6d). Histopathological analysis revealed no significant tissue damage in the heart, lung, liver, spleen, and kidney in all groups. Collectively, these results demonstrated that Au@Bi_2_S_3_ nanoreactors exhibited satisfactory biocompatibility and biosafety in vivo. In order to evaluate the long-term toxicity of Au@Bi_2_S_3_ nanoreactors in vivo, we added key parameters for blood biochemistry testing after 14-day treatment period, including liver function indexes (ALT, AST), renal function indicators (creatinine, urea), and inflammation cytokines IL-6 and TNF-α. Biochemical analyzer was used to detect ALT, AST, creatinine and urea. The results showed that compared with normal mice, the overall biochemical indexes of mice injected with Au@Bi_2_S_3_ were slightly higher, but there was no statistical difference, which indicated that the nanoreactors hardly affected the liver and kidney functions of mice (Fig. S28a–d). ELISA was used to detect the expression of TNF-α and IL-6 in serum of mice. The results showed that there was no statistical difference in the expression of related inflammatory indexes between the nanoreactors group and normal mice group (Fig. S28e–f). In addition, the optical in vivo imaging technology was used to detect the accumulation of the Au@Bi_2_S_3_ nanoreactors in liver and kidney at different time points. The results showed that Au@Bi_2_S_3_ nanoreactors were metabolized in liver and kidney, and accumulated obviously after 6 h, but were obviously metabolized after 24 h, and almost completely metabolized after 48 h (Fig. S30). Combined with the analysis of the results of serum biochemical indexes of mice treated for 14 days, the Au@Bi_2_S_3_ nanoreactors had almost no damage to liver and kidney. These results confirmed the biological safety of Au@Bi_2_S_3_. The above results showed that the nanoreactors had good biocompatibility and provides feasibility for its clinical application.

### 3.5 Therapeutic effect in vivo

Au@Bi_2_S_3_ has shown promising anti-tumor effects in vitro and good biosafety in vivo, suggesting its potential for further application in therapeutic studies. To better elucidate the interaction between the tumor and host, and simulate clinical characteristics, the therapeutic effect of Au@Bi_2_S_3_ was directly observed in vivo using a subcutaneous tumor xenograft model with HCC cells. The schematic diagram of model construction and processing was established (Fig. 6a). PBS, Bi_2_S_3_, or Au@Bi_2_S_3_ were injected into the tumor at a dose of 150 μL (1 mg/mL). The tumor areas in three phototherapy groups were exposed to light (λ > 420 nm) for 30 min after injection. By evaluating the tumor size after treatment, it has been found that the effect of inhibiting tumor growth when treated with Au@Bi_2_S_3_ under light was the most significant (Fig. 6c and e). Next, we detected the expression levels of PCNA, Bax, Bcl-2, CDK2, and CDK6. The results revealed that the expression levels of PCNA, CDK2, and CDK6 distinctly decreased in the treatments with Bi_2_S_3_ and Au@Bi_2_S_3_ under light. The expression level of Bax was also elevated to some extent, while the expression level of Bcl-2 was decreased. Moreover, the degree of change in the treatment with Au@Bi_2_S_3_ surpassed the treatment with Bi_2_S_3_ under light (Fig. 6f). Next, H&E staining showed that tissue damage was significantly improved in both treatments with Au@Bi_2_S_3_ and Bi_2_S_3_ under light, especially in the treated with Au@Bi_2_S_3_. Meanwhile, immunohistochemical results demonstrated that the expressions of E-cadherin and N-cadherin (markers of epithelial-mesenchymal transition (EMT) in tumor) were decreased (Fig. 6i). These findings suggested that these composite nanoreactors could mitigate the progression of HCC under phototherapy conditions, with Au@Bi_2_S_3_ nanoreactors demonstrating a more pronounced therapeutic effect. In addition to the above in vivo animal experiments, we also introduced the use of HCC organoids. Organoids, the collection of organ-specific cell types developed from stem cells or organ progenitor cells, can simulate the tissue structure and function in vivo to the greatest extent. The therapeutic effect of the Au@Bi_2_S_3_ nanoreactors on HCC was further verified at the organoids level. The construction of HCC organoids was shown in Fig. 6g. H&E staining result showed that the treatment of Au@Bi_2_S_3_ nanoreactors reduced the efficiency and size of organoids under light, which supported that the nanoreactors intervened in the formation and maintenance of organoids. Immunohistochemical results of AFP and Heppar-1 showed that the constructed organoids were HCC organoids. The proliferation index Ki-67 decreased significantly for Au@Bi_2_S_3_ under light, indicating that this treatment could inhibit the proliferation of HCC organoids (Fig. 6h).

### 3.6 Au@Bi_2_S_3_ nanoreactors activated the Hippo/Yap signaling pathway

**Fig. 7:**
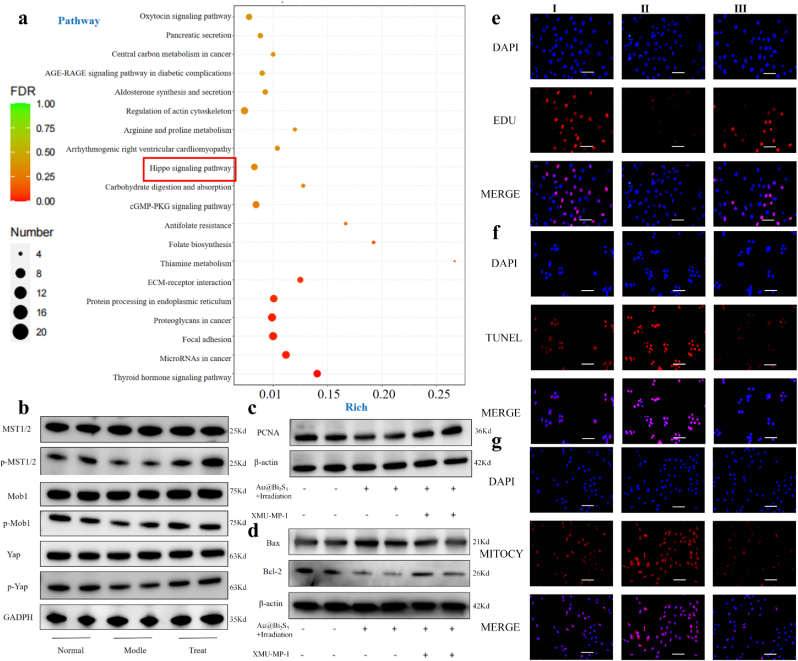
a): RNA sequencing was performed on HepG-2 cells treated with Au@Bi_2_S_3_ and untreated cells to analyze enrichment maps of pathway changes; b):The changes of protein levels related to the Hippo/Yap signaling pathway were detected by using Western blotting; c): Western blotting detection of changes in proliferative protein PCNA in three different treatments groups; d): Western blotting was used to detect the expression of apoptosis-related proteins Bax and Bcl-2; e): EDU staining was used to detect the proliferation of HepG-2 cell; f): TUNEL staining was used to detect of the apoptosis of HepG-2 cell; g): The Mito-Tracker Deep Red FM probe was used to detect the release of ROS in HepG-2 cells. Ⅰ: control; Ⅱ: Au@Bi_2_S_3_ under light; Ⅲ: Au@Bi_2_S_3_ under light + XMU-MP-1.

To unravel the underlying mechanism of Au@Bi_2_S_3_ in HCC cells, we conducted the RNA-sequencing analysis on HepG-2 cells. The KEGG pathway enrichment analysis of the RNA sequencing data revealed the emergence of the Hippo signaling pathway (Fig. 7a). It suggested that the Hippo/Yap signaling pathway played a role in the development of HCC. The Hippo/Yap signaling pathway functions as a critical regulator of hepatic growth and tumor suppression in the mammalian liver [49]. In the mouse liver, hippopotamus kinases Ste20-like kinases 1/2 (MST1/2) exert inhibitory effects on hepatocyte proliferation, survival, and hepatocyte formation by suppressing the activation of Yes-associated protein/Transcriptional coactivator with PDZ-binding motif (Yap/TAZ) [50].

Kinases MST1/2 are the closest mammalian homologues of drosophila melanogaster kinase Hippo [51,52]. The key kinases MST1/2 of the Hippo signaling pathway are also closely related to ROS. Studies have shown that kinases MST1 and MST2 positively regulate the ROS induction and bactericidal activity of phagocytes [53]. Consistent with the above results, Western blotting results confirmed that there were no significant changes in the protein levels of Mob1, Yap, and MST1/2, while the expression levels of p-Mob1, p-Yap, and p-MST1/2 were decreased in the tumor model and elevated in the treatment with Au@Bi_2_S_3_ (Fig. 7b). XMU-MP-1 is an inhibitor of the Hippo/Yap signaling pathway, capable of significantly inhibiting this signaling pathway. The Western blotting results showed that treatment of HepG-2 cells with XMU-MP-1 increased the levels of PCNA and Bcl-2, while it decreased Bax expression. XMU-MP-1 could inhibit HepG-2 cell apoptosis induced by Au@Bi_2_S_3_ (Fig. 7c and d). Furthermore, the results from EDU staining and colony formation experiments suggested that the application of XMU-MP-1 enhanced cell proliferation in the treatment with Au@Bi_2_S_3_ (Fig. 7e–S22b). The TUNEL staining and flow cytometry-based apoptosis assay revealed a reduction in the apoptotic rate in the inhibitor-treated group (Fig. 7f–S22a). Transwell results indicated that the inhibitor further enhanced migration and invasion (Fig. S23). The above results showed that the Hippo/Yap signaling pathway inhibitor notably impaired the therapeutic efficacy compared to the treatment group.

### 3.7 Au@Bi_2_S_3_ nanoreactors promoted ROS production

Gold nanoparticles can accumulate more ionizing radiation energy and stimulate the generation of photoelectrons and Auger electrons during radiotherapy. Photoelectrons and Auger electrons can interact with water and oxygen to produce active free radicals that cause cell damage and radiosensitization effect. Moreover, Bi element with high atomic number also has excellent radiosensitization performance [54,55]. The reactive and physiologically harmful free radicals, ROS, are mainly formed in mitochondria and are involved in the regulation of different physiological processes, including apoptosis and autophagy [[56], [57], [58], [59], [60], [61]]. To detect the release of ROS, two kinds of ROS probes including DHE and Mito-Tracker Deep Red FM, were used in HepG-2 cells. The results showed that the release of ROS was promoted when treated with Au@Bi_2_S_3_ under light compared with other treatments (Fig. 5e and f). In addition, after XMU-MP-1 inhibited the Hippo/Yap signaling pathway, the release of ROS was decreased (Fig. 7g–S24). To verify mitochondrial structural damage at the cellular level, the TEM was used to observe the structure of mitochondrion in HepG-2 cells. The results of TEM revealed stark contrasts between experimental and control groups (Fig. S25). In untreated cells, mitochondria maintained intact elliptical profiles with densely packed, uniformly organized cristae. Conversely, cells treated with Au@Bi_2_S_3_ and light irradiation exhibited severe mitochondrial ultrastructural disruption, characterized by cristae fragmentation (partial to complete loss of cristae architecture) and extensive matrix vacuolization. These findings corroborated the that the nanoreactors induced massive ROS production leading to mitochondrial disruption.

**Fig. 8:**
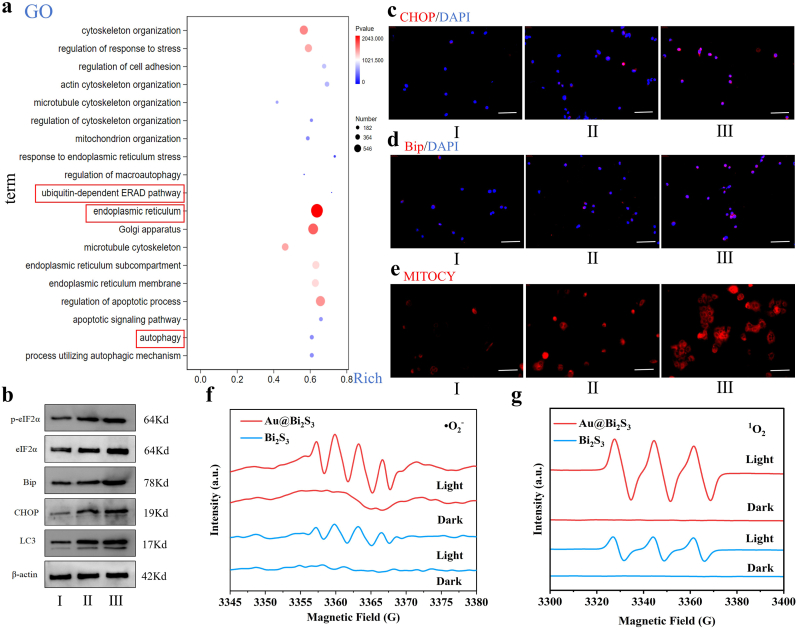
a): RNA sequencing was performed on HepG-2 cells treated with Au@Bi_2_S_3_ and untreated cells to generate enrichment maps of functional changes; b): Western blotting was used to detect the changes in protein levels related to the ER stress-related proteins including eIF2α, endoplasmic reticulum folding-related proteins Bip, CHOP and autophagy-related proteins LC3; c): The changes of CHOP were detected by immunofluorescence; d): The changes of Bip were detected by immunofluorescence; e): The Mito-Tracker Deep Red FM probe was used to detect the release of ROS in HepG-2 cells; f): EPR spectra of Bi_2_S_3_ and Au@Bi_2_S_3_ was used to measure •O_2_^−^; g): EPR spectra of Bi_2_S_3_ and Au@Bi_2_S_3_ was used to measure ^1^O_2_. Ⅰ: control; Ⅱ: Bi_2_S_3_ under light; Ⅲ: Au@Bi_2_S_3_ under light.

In addition, the GO function enrichment analysis found that the function of ER stress was activated and autophagy increased under the treatment of Au@Bi_2_S_3_ accompanied by light (Fig. 8a). The ER is the central organelle for the secretion, synthesis, folding, and modification of transmembrane proteins [62]. Protein treatment, modification, and folding in ER are strict regulatory processes that determine the function, fate and survival of cells [63]. Environmental stressors such as nutritional deficiency, hypoxia, ROS accumulation, and acid-base imbalance will challenge the functional integrity of the ER [[64], [65], [66]]. After various factors lead to ER stress, elF2α phosphorylation increases, inhibiting normal protein translation. Acute ER stress typically results in enhanced antioxidant defense, reduced oxidative stress (lower ROS levels), and increased cell survival. In contrast, persistent ER stress can lead to elevated protein synthesis, heightened oxidative stress (increased ROS levels), and the promotion of apoptosis. Then, to verify this discovery, we observed that treatment with nanoreactors led to increased expression of ER stress-related proteins. Specifically, the expression of the persistent ER stress protein CHOP was elevated, and there was also an enhancement in the expression of autophagy-related proteins, indicating increased autophagy (Fig. 8b). At the same time, the results of immunofluorescence also showed the same results (Fig. 8c and d). Significantly, the production of ROS was increased (Fig. 8e). To further prove the generation of ROS, electron paramagnetic resonance (EPR) was used to determine the generation and type of ROS.

Because the redox potential of •OH produced by water oxidation is 2.73 V vs. RHE, which is much higher than the VB potential of bismuth sulfide (1.48 V vs. RHE), Bi_2_S_3_ and Au@Bi_2_S_3_ do not produce •OH signal in dark and light (Fig. S14). In addition, the 9,10-anthracenediyl-bis (methylene) dimalonic acid (ABDA) probe was used to explore the influence of ^1^O_2_. This is a specific detection probe for ^1^O_2_. The results showed that there was the significant ABDA degradation by Au@Bi_2_S_3_ with light (90.9 % vs. 63.9 % for Bi_2_S_3_) (Fig. S26). This result confirmed Au@Bi_2_S_3_ with light could generate ^1^O_2_.

Furthermore, the EPR results (Fig. 8f and g) showed **·**O_2_^−^ and ^1^O_2_ generation. These results demonstrated that Au@Bi_2_S_3_ operated via **·**O_2_^−^ and ^1^O_2_ pathways, and had a critical advantage over conventional photosensitizers.

After dark adsorption for 30 min, illumination was carried out for 75 min. The degradation rate of methylene blue (MB) with a concentration of 10 mg/L by 30 mg/mL of Au@Bi_2_S_3_ was 73.5 %, while the degradation rate of MB by Bi_2_S_3_ at the same concentration was only 53.6 %. In addition, first-order kinetics showed that compared with Bi_2_S_3_, the reaction rate constant of MB degradation by Au@Bi_2_S_3_ increased, indicating that Au@Bi_2_S_3_ can generate more ROS and has a higher ROS generation efficiency (Fig. S15). To investigate the photothermal properties of Au@Bi_2_S_3_, we used an infrared thermal imager to record the temperature changes of Bi_2_S_3_ and Au@Bi_2_S_3_ during illumination. At a concentration of 64 μg/mL, the temperature stabilized after 6 min of illumination. The maximum temperatures of both Bi_2_S_3_ and Au@Bi_2_S_3_ were below 42 °C, indicating that the photothermal effect was insignificant (Fig. S16). Combined with the previous experimental results, the treatment of Au@Bi_2_S_3_ could strengthen the ER stress of HepG-2 cells, promote ROS production and autophagy, lead to HCC cell apoptosis and inhibit the progress of tumor.

The above results showed that Au@Bi_2_S_3_ with good biological safety has a good anti-tumor effect in vitro and in vivo, as well as in organ-like models, which may have the transformation potential to clinical application. PEGylation, a clinically validated strategy for improving nanomedicine pharmacokinetics, could ensure biocompatibility and safety when optimized. Our results confirmed minimal toxicity of Au@Bi_2_S_3_ in vitro and in vivo (Fig. 4, Fig. 6b, S21). AuNPs are FDA-approved for rheumatoid arthritis therapy (e.g., Aurim™) [68], while Au@Bi_2_S_3_ is under clinical exploration as a CT contrast agent [69], collectively supporting translational feasibility. The nanoreactors could enable precision HCC therapy through Schottky junction-driven, ROS generation. Its diagnostic function and extensible synthetic position make it a potential paradigm shift platform for solid tumor treatment.

## 4 Conclusion

In conclusion, we developed the Au@Bi_2_S_3_ nanoreactors with a Schottky junction that significantly enhances PDT efficacy against HCC. The heterojunction structure facilitates efficient electron-hole separation, doubling ROS generation compared to Bi_2_S_3_ alone. The Au@Bi_2_S_3_ composite markedly suppressed the growth of transplanted tumors in vivo and hindered the proliferation of HCC organs at organoid level. The high levels of ROS produced by Au@Bi_2_S_3_ nanoreactors activated the Hippo/Yap signaling pathway, enhanced ER stress and autophagy in tumor cells, leading to increased apoptosis. This work provides a blueprint to design and synthesize efficient photodynamic nanoreactors, bridging nanotechnology and molecular oncology to address unmet clinical needs.

## CRediT authorship contribution statement

**Liangsong Tao:** Writing – original draft, Validation, Methodology. **Rongrong Gu:** Writing – original draft, Validation, Methodology. **Junfa Yang:** Writing – original draft, Validation, Methodology. **Jiewei Wang:** Writing – original draft, Validation, Methodology. **Tiling Wu:** Writing – original draft, Validation, Methodology. **Xianyue Rao:** Writing – original draft. **Hao Wang:** Writing – review & editing. **Cheng Qian:** Software, Funding acquisition. **Jian Liu:** Writing – review & editing. **Sheng Ye:** Writing – review & editing, Funding acquisition. **Tao Xu:** Writing – review & editing, Funding acquisition.

## Ethics statement

All animal testing procedures have been approved by the ethical guidelines and reviewed and implemented in accordance with the standards of the Experimental Animal Ethics Committee of the First Affiliated Hospital of USTC (2023-N (A) −78).

## Declaration of competing interest

The authors declare that they have no known competing financial interests or personal relationships that could have appeared to influence the work reported in this paper.

## Data Availability

Data will be made available on request.
