# Supplementary material for: Schottky barrier in pea-like Au@Bi2S3 nanoreactor enabling efficient photodynamic therapy of hepatocellular carcinoma
Source: Mater Today Bio. 2025 Jun 19;33:102001. doi: 10.1016/j.mtbio.2025.102001 (PMC12241386; doi:10.1016/j.mtbio.2025.102001)
Supplement: Multimedia component 1 [file mmc1.docx]

Supporting Information

Schottky Barrier in Pea-like Au@Bi_2_S_3_ Nanoreactor Enabling Efficient Photodynamic Therapy of Hepatocellular Carcinoma

Liangsong Tao^a, 1^, Rongrong Gu^b, 1^, Junfa Yang^c, 1^, Jiewei Wang^b, 1^, Tiling Wu^d^, Xianyue Rao^d^, Hao Wang^d, *^, Cheng Qian^e^, Jian Liu^f, *^, Sheng Ye^b, *^ and Tao Xu^a,^ ^*^

*^a^ Inflammation and Immune Mediated Diseases Laboratory of Anhui Province, Anhui Institute of Innovative Drugs, School of Pharmaceutical Sciences, Anhui Medical University, Hefei, 230032, China, Institute for Liver Diseases of Anhui Medical University*

*^b^* *Agricultural Photocatalysis Laboratory, School of Materials and Chemistry, Anhui Agricultural University, Hefei 230036, China*

*^c^* *Department of Emergency Surgery, the Second Affiliated Hospital of Anhui Medical University, Hefei 230601, China*

*^d^ Department of Radiation Oncology, The First Affiliated Hospital of Anhui Medical University, Hefei 230022, China*

*^e^ Center for Scientific Research, Anhui Medical University, Hefei 230032, China*

*^f^ College of Chemistry and Chemical Engineering, Inner Mongolia University, Hohhot 010021, China*

^⁎^ Correspondence authors.

E-mail: xutao@ahmu.edu.cn (T. Xu), sye503@ahau.edu.cn (S. Ye), jian.liu@surrey.ac.uk (J. Liu), wanghao@ahmu.edu.cn (H. Wang).

^1^ These authors contributed equally to this work.

**Experimental Section**

*X-ray Diffraction (XRD):* The prepared sample was flattened and attached to the sample stage for testing. The scanning angle ranged from 10° to 80°, with a scanning speed of 5°/min and a scanning step size of 0.02°.

*Scanning Electron Microscopy (SEM):* The sample powder was directly adhered to the surface of conductive adhesive for analysis. During testing, the accelerating voltage was set within the range of 0.5-30 kV.

*Transmission Electron Microscopy (TEM):* The powder sample was dispersed in absolute ethanol via ultrasonic treatment. An appropriate amount of the dispersion was then dropped onto a copper grid. The accelerating voltage during testing was set at 80 kV.

*X-ray Photoelectron Spectroscopy (XPS):* The sample was affixed to a copper sheet, and loose powder on the surface was blown away before placing it on the test stage.

*Electron Paramagnetic Resonance (EPR):* The sample was dissolved in methanol or water, and an appropriate amount of free radical scavenger (TEMPO/DMPO) was added. After thorough mixing, the EPR sample tube was sealed and placed in the pre-set EPR spectrometer.

*Fourier Transform Infrared Spectroscopy (FTIR):* A Nicolette is 50 FTIR spectrometer (Thermo Fisher Scientific, USA) was used. The test was conducted in attenuated total reflection (ATR) mode, covering a range of 650–4000 cm^-1^.

*Ultraviolet-Visible Spectroscopy (UV-Vis):* The Au@Bi_2_S_3_ powder was pressed into a thin, translucent sheet on a gasket using a quartz rod. Uniform spin-coating is preferred to ensure a smooth surface, as excessive roughness may hinder signal acquisition or reduce the signal-to-noise ratio.

*Photoelectrochemical test:* Using a saturated calomel electrode as the reference electrode, a platinum sheet as the counter electrode, and the studied material as the working electrode, the photocurrent response and ELS curves were measured under an electrochemical workstation (Wuhan Corrtest CS310M) and a xenon lamp light source (Beijing MerryChange Technology Co.,Ltd. MC-PF300C).

*Cell migration and invasion assays:* Transwell chambers (Corning, Inc.) were used to evaluate cell invasion with inserts precoated with Matrigel (1 mg/mL) at 37 °C for 30 min. HCC cells were resuspended in high-glucose DMEM containing 1% FBS and seeded into the upper Transwell chamber at a density of 1×10^5^ cells/well and 500 μL of high-glucose DMEM containing 10% FBS was added to the corresponding lower chamber. For the migration assay, Transwell chambers (Corning, Inc.) without inserts precoated with Matrigel were used, otherwise, the same protocol as for the invasion assay was followed. After incubation for 48 hours at 37 °C, the Transwell chambers were fixed with 4% paraformaldehyde at room temperature for 30 min and stained with 0.5% crystal violet at room temperature for 20 minutes. Finally, the number of stained cells in the lower chamber was counted manually in each field and images were captured under a light microscope (magnification, ×200; Olympus Corporation).

*Flow cytometry:* A suitable number of cells were taken and inoculated in a six-well plate. Before light, cells in each group were washed twice with PBS, and then the apoptotic cells in PBS were discarded. After processing the corresponding treatments mentioned above, cells were collected. The supernatant and digested cells were combined and centrifuged. Cells were washed twice with precooled PBS, and then 100 μL of 1× binding buffer was added to each tube immediately followed by staining with 5 μL Annexin V-FITC (Becton-Dickinson, BD; San Jose, CA, USA) and 5 μL PI (BD). After incubating at room temperature for 15 minutes in the dark, 200 μL of 1×binding buffer was added and mixed, the cell suspension was transferred to a 5 mL flow cytometer in the dark, and testing on the flow cytometer was performed within 1 hour.

*Western blotting:* Tissues or cells were collected after being given different treatments. Then the electrophoresis of cell protein samples on the SDS-PAGE gel was conducted, and the samples were transferred to a PVDF membrane and incubated at room temperature with 5% nonfat dry milk for 1 hour. The membrane was treated with primary antibodies and incubated overnight at 4 °C. The following antibodies were used: PCNA (CST, 1: 1000), Bcl-2 (CST, 1: 1000), Bax (CST, 1: 1000), CDK2 (CST, 1: 1000), CDK6 (CST, 1: 1000), and β-actin (Yeasen, 1: 5000). MST1/2 (CST, 1:2000), p-MST1/2 (CST, 1: 2000), Mob1 (CST, 1:2000), p-Mob1 (CST, 1: 2000), Yap (CST, 1:2000), p-Yap (CST, 1: 2000), MST1/2 (CST, 1:2000), p-MST1/2 (CST, 1: 2000), eIF2α (CST, 1: 2000), p-eIF2α (CST, 1:2000), CHOP (1: 1000), Bip (CST, 1: 1000), LC3 (CST, 1: 1000). After 1-hour incubation with the secondary antibodies (Jackson Lab, 1: 10,000), protein bands were visualized using the ECL Western blotting Detection Reagent (Tanon, Shanghai, China) and quantified using the Gel-Pro Analyzer (Tanon, Shanghai, China).

*Real-time Reverse Transcription Polymerase Chain Reaction (RT-qPCR):* Cultured cells were homogenized using TRIzol reagent (Invitrogen). Following reverse transcription, we conducted quantitative mRNA expression analysis using the SYBR Green Master Mix by the manufacturer’s protocol. Fold changes in mRNA levels of target genes were normalized to the constant control GAPDH. The PCR primer sequences involved were designed in Table 1.

**Table 1.** The PCR primer sequences

| Gene | Primer pair | |
| --- | --- | --- |
| Bax | F:5′-CCCGAGAGGTCTTTTTCCGAG-3′ | R:5′-CCAGCCCATGATGGTTCTGAT-3′ |
| Bcl-2 | F:5′-GGTGGGGTCATGTGTGTGG-3′ | R:5′-CGGTTCAGGTACTCAGTCATCC-3′ |
| PCNA | F:5′-ACACTAAGGGCCGAAGATAACG-3′ | R:5′-GGCTGTTGTCATACTTCTCATGG-3′ |
| GAPDH | F:5′-GGAGCGAGATCCCTCCAAAAT-3′ | R:5′-ACAGTCTTCTGAGTGGCAGTGA-3′ |

*Immunohistochemistry:* Tumor tissues were fixed in 4% neutral paraformaldehyde, followed by routine histological staining. The tissues were then embedded in paraffin, and the sections were dewaxed and rehydrated. Antigen retrieval was performed by microwaving in sodium citrate for 15 minutes. After washing the tissue sections three times with PBS, they were blocked and incubated with the primary antibody overnight at 4 °C. Subsequently, the sections were incubated with the diluted secondary antibody. Finally, the slices underwent color development, counterstaining, and mounting.

*Construction of HCC organoids:* Tissue samples were taken from the ultra-clean table in Petri dishes (solid tumor tissue samples (during operation) were obtained from patients by professional medical personnel in professional medical institutions, and all patients signed informed consent forms. During the operation, the sample was 0.25 cm^3^, which was stored and transported by commercial tissue preservation solution), the blood-bearing tissue was removed, washed twice with lotion (PRS-TCR-1), and the tissue was transferred to another Petri dish for mechanical separation with a sterile scalpel, and the tissue block was divided into 1×1×1 mm^3^. Suck the cut intraoperative tissue into a 15 mL centrifuge tube, add 5 mL lotion, mix well, and centrifuge at 1500 rpm for 4 minutes; Discard the supernatant, add digestive enzyme diluent (PRS-TDD-2) and tissue digestive enzyme (PRS-TDE-2) in a ratio of 1: 3, mark the name and number of the sample, seal it with a sealing film, and digest it in a shaker at 300 rpm (Zhichu Instrument ZQLY-180N) at 37 ℃. Observe whether the digestion is completed every 30 minutes, and judge whether it is naked. After digestion, the undigested tissue mass was filtered through a 100 µm filter screen, and the tissue mass on the filter screen was washed into a centrifuge tube with lotion to reduce cell loss, and centrifuged at 1500 rpm at 25 ℃ for 4 minutes. Discard the supernatant, and observe whether there are blood cells. If there are blood cells, add 8 mL of blood cell lysate (purchased from Sigma Company), mix them evenly, crack them at 4 ℃ for 20 minutes, mix them upside down once, and centrifuge at 1500 rpm at 25 ℃ for 4 minutes. The supernatant was discarded, and the heavy suspension cells were cultured in DF12. A small amount of heavy suspension cells were transferred and laid flat in a Petri dish, and the density and morphology of cancer cells were observed under a microscope (CNOPTEC, BDS400).

*The ABDA probe:* Bi_2_S_3_ and Au@Bi_2_S_3_ nanoreactors (64 μg/mL) were dispersed in PBS containing 50 μM ABDA (9,10-anthracenediyl-bis (methylene) dimalonic acid) and incubated in the dark for 30 minutes with continuous stirring. The suspension was irradiated using a full-spectrum light source equipped with a 420 nm cutoff filter at an intensity of 0.4 W/cm^2^. Absorbance at 378 nm (OD_378_) was measured at 5-minute intervals over 30 minutes. The singlet oxygen (^1^O_2_) generation rate was calculated based on ABDA degradation percentage. A solution containing ABDA alone (no nanoreactors) was subjected to identical irradiation conditions to account for background photolysis.

*Construction of hypoxic environment:* To rigorously evaluate the hypoxia-resilient PDT efficacy of Au@Bi_2_S_3_, a comprehensive experimental framework was established. HepG-2 cells were cultured under hypoxic conditions (1% O_2_, 5% CO₂, 94% N₂) for 24 hours using a modular hypoxia chamber, with parallel validation via chemical hypoxia induction using 200 μM CoCl₂

The TRPL data can be well fitted by a biexponential function of time (t):

$$I\left( t \right)=A_{1}e^{-\frac{1}{\tau1}}+A_{2}e^{-\frac{1}{\tau2}}+I_{0}$$

According to the fitting results, the average composite life $\tau_{\mathrm{avg}}$ is calculated as follows:

$$\tau_{avg}=\frac{{(A}_{1}\tau_{1}^{2}+A_{2}\tau_{2}^{2})}{{(A}_{1}\tau_{1}+A_{2}\tau_{2})}$$

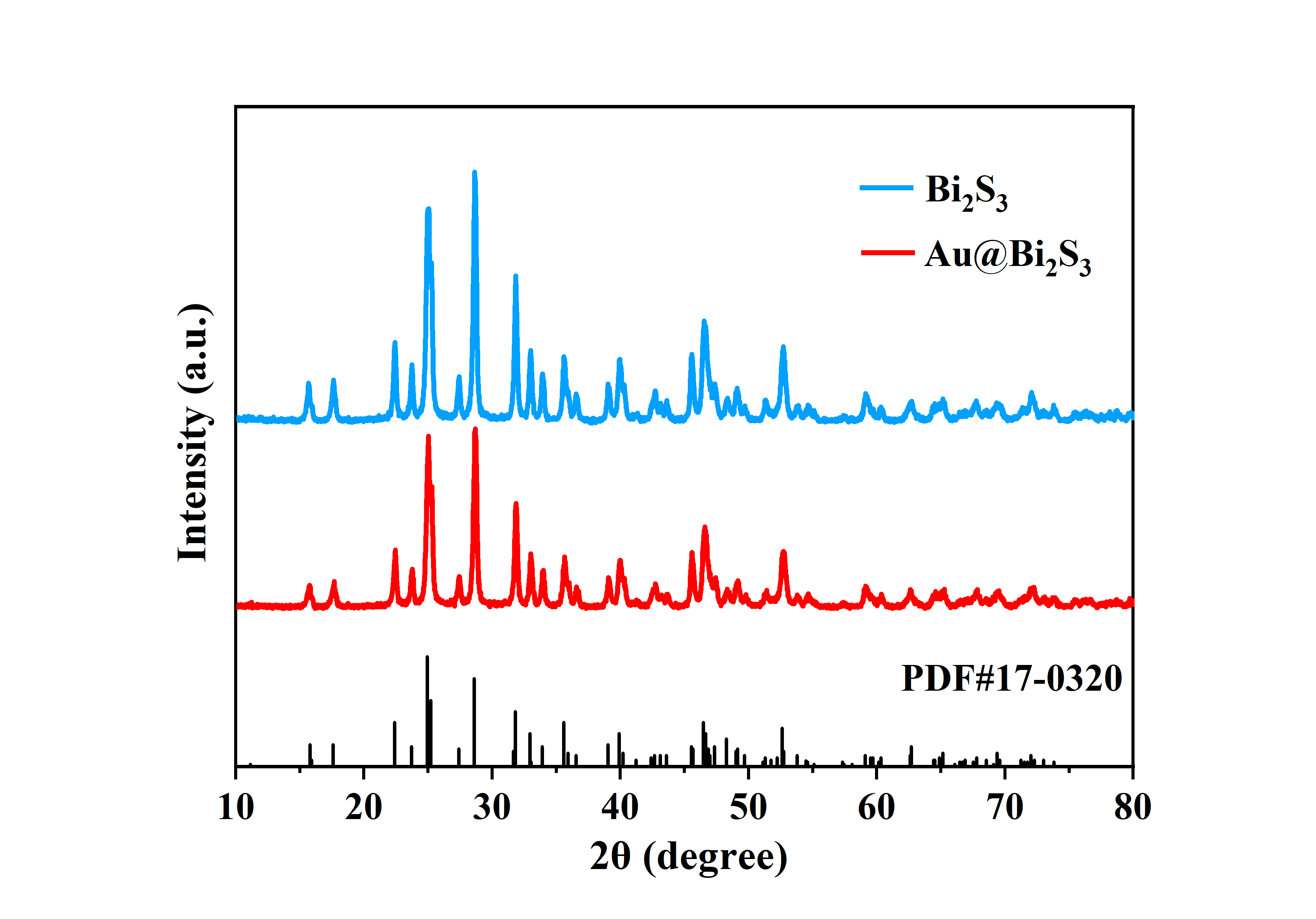


**Figure S1.** The XRD of Bi_2_S_3_ and Au@Bi_2_S_3_ nanoreactors, there was no change in XRD spectra before and after the introduction of Au nanoparticles.


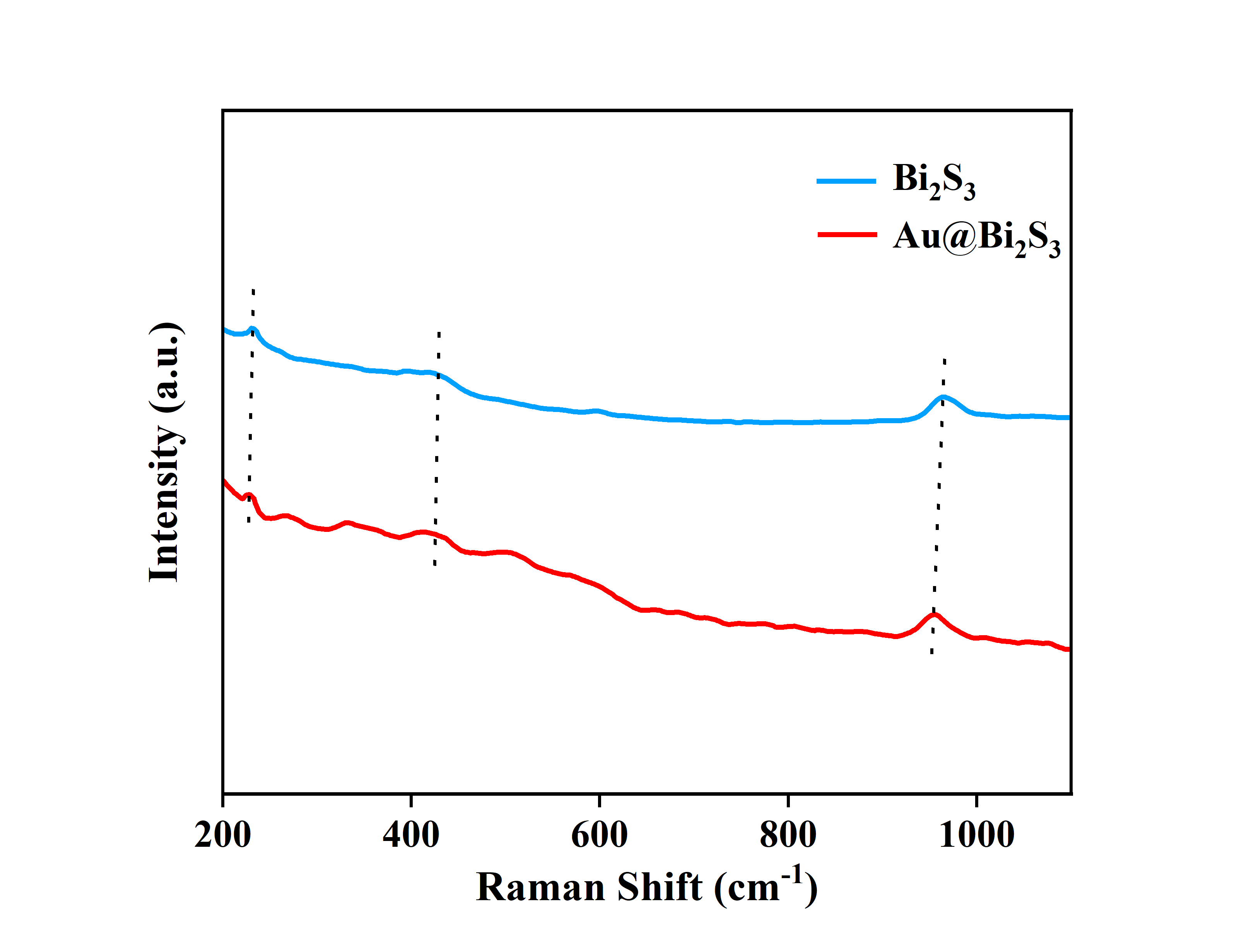


**Figure S2.** The Raman spectrum of Bi_2_S_3_ and Au@Bi_2_S_3_ nanoreactors, Raman spectroscopy reveals peaks at 232, 430, and 967 cm^-1^.


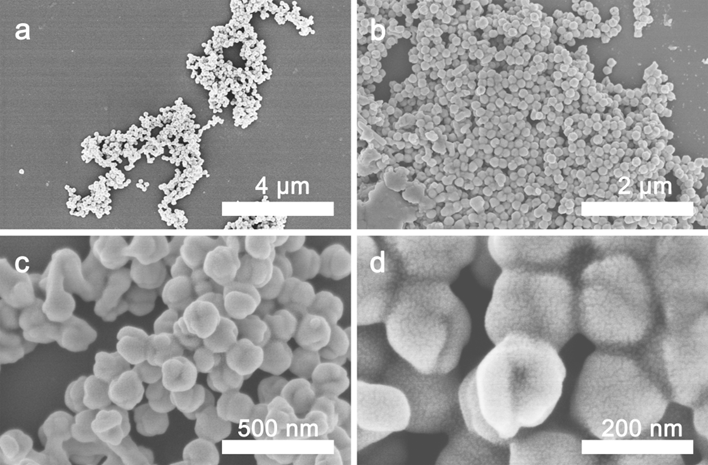


**Figure S3.** a-d): SEM images of Bi_2_S_3_ nanoreactors at different magnifications.


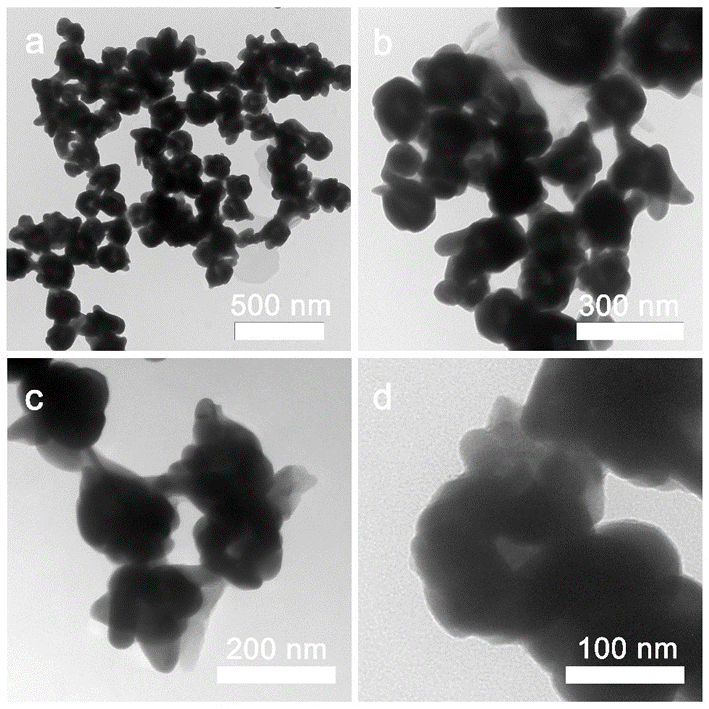


**Figure S4.** a-d): TEM images of Bi_2_S_3_ nanoreactors at different magnifications.


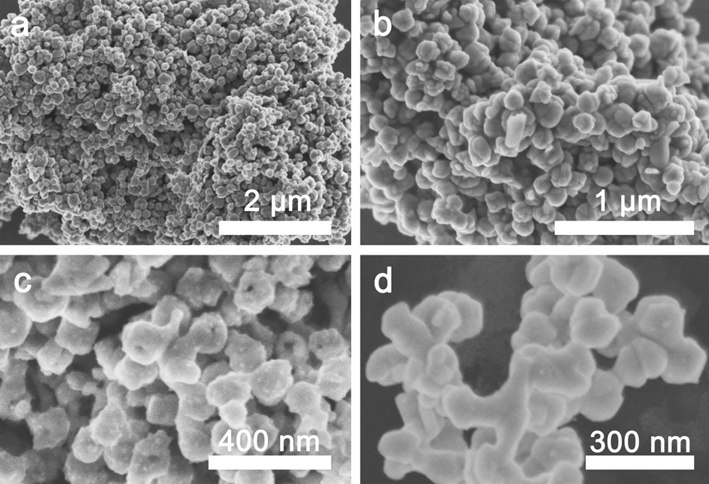


**Figure S5.** a-d): SEM images of Au@Bi_2_S_3_ nanoreactors at different magnifications.


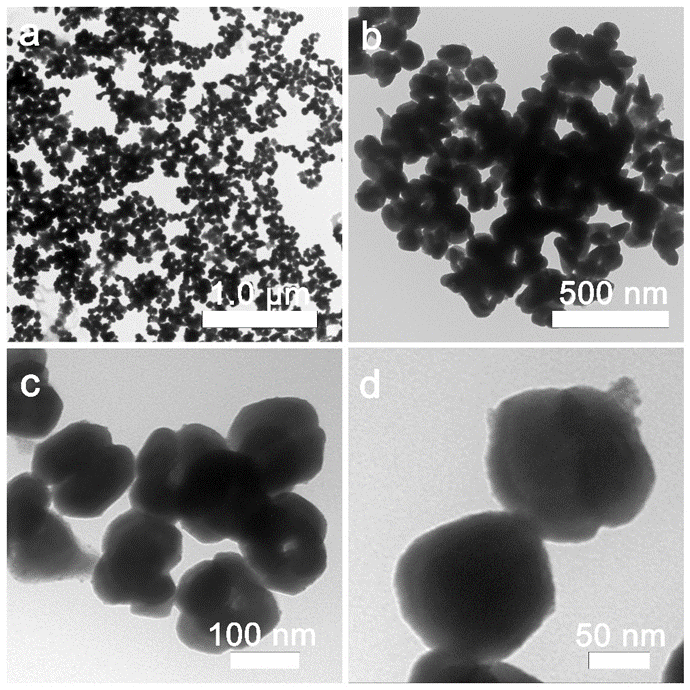


**Figure S6.** a-d): TEM images of Au@Bi_2_S_3_ nanoreactors at different magnifications.


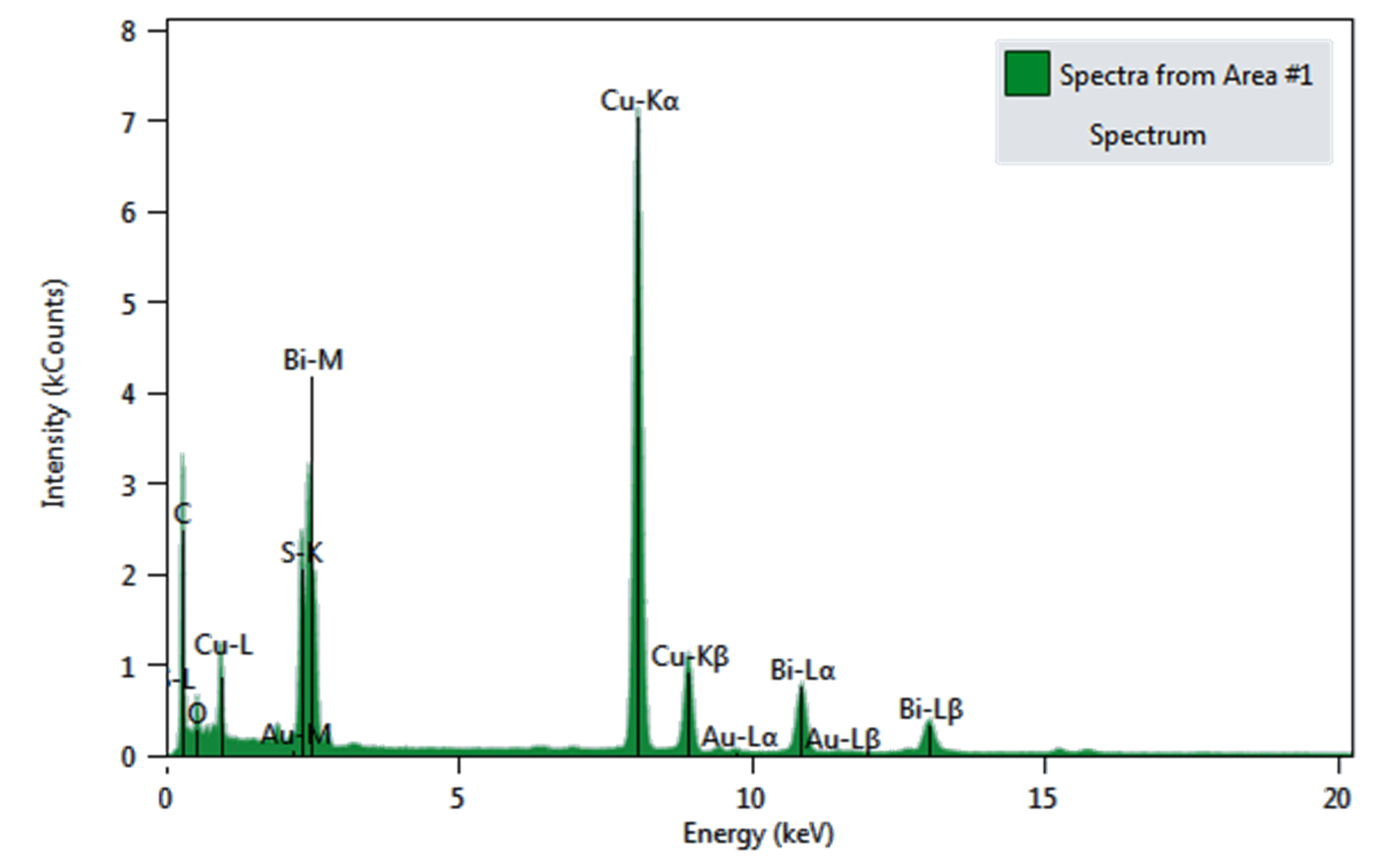


**Figure S7.** Energy-dispersive spectroscopy (EDS) of Figure 2e-h. The result of EDS showed that the element distribution in the Au@Bi_2_S_3_ contained Au element.


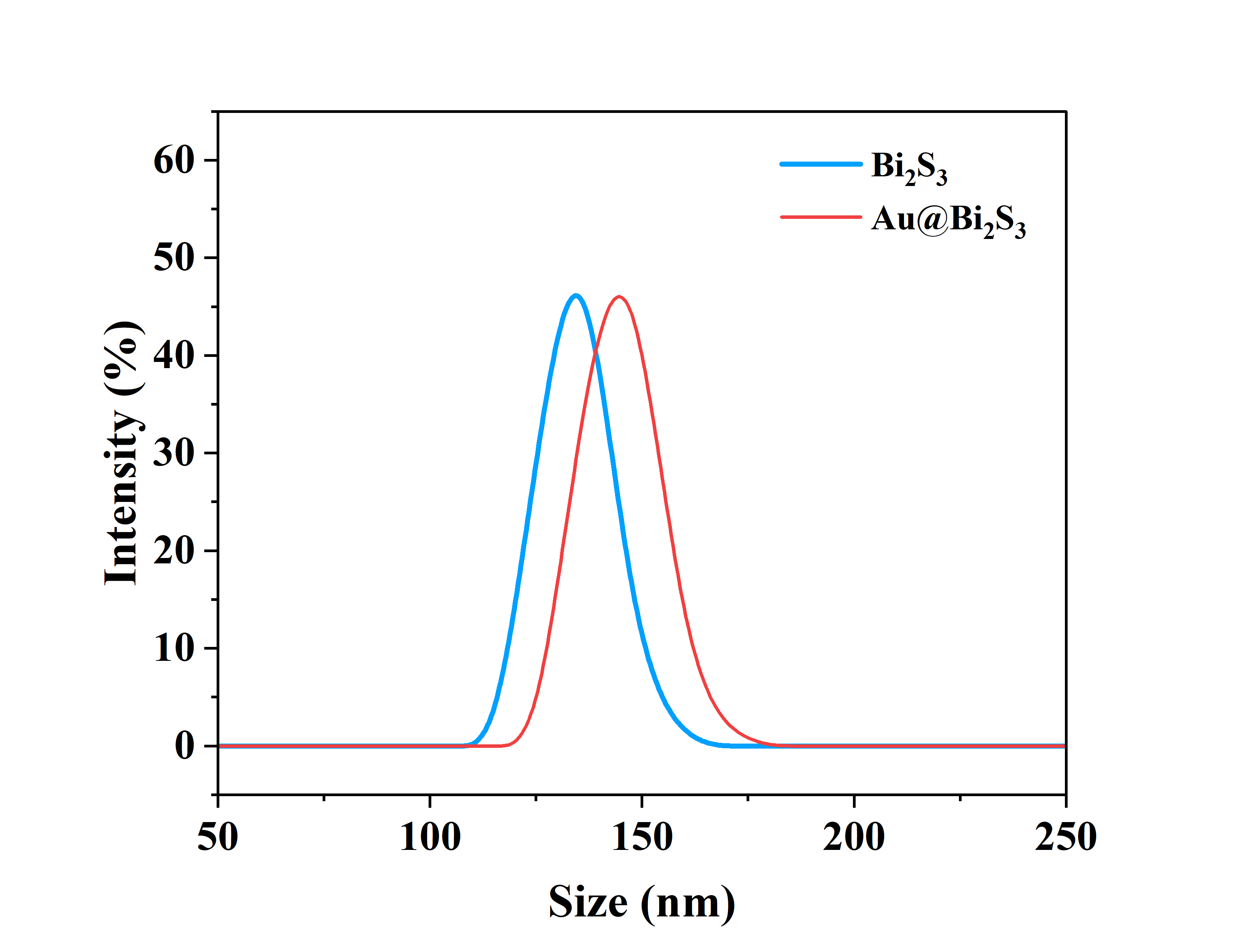


**Figure S8.** The dynamic light scattering (DLS) particle size distribution of Bi_2_S_3_ and Au@Bi_2_S_3_, the particle sizes of Bi_2_S_3_ and Au@Bi_2_S_3_ are approximately 140 and 150 nm, respectively.


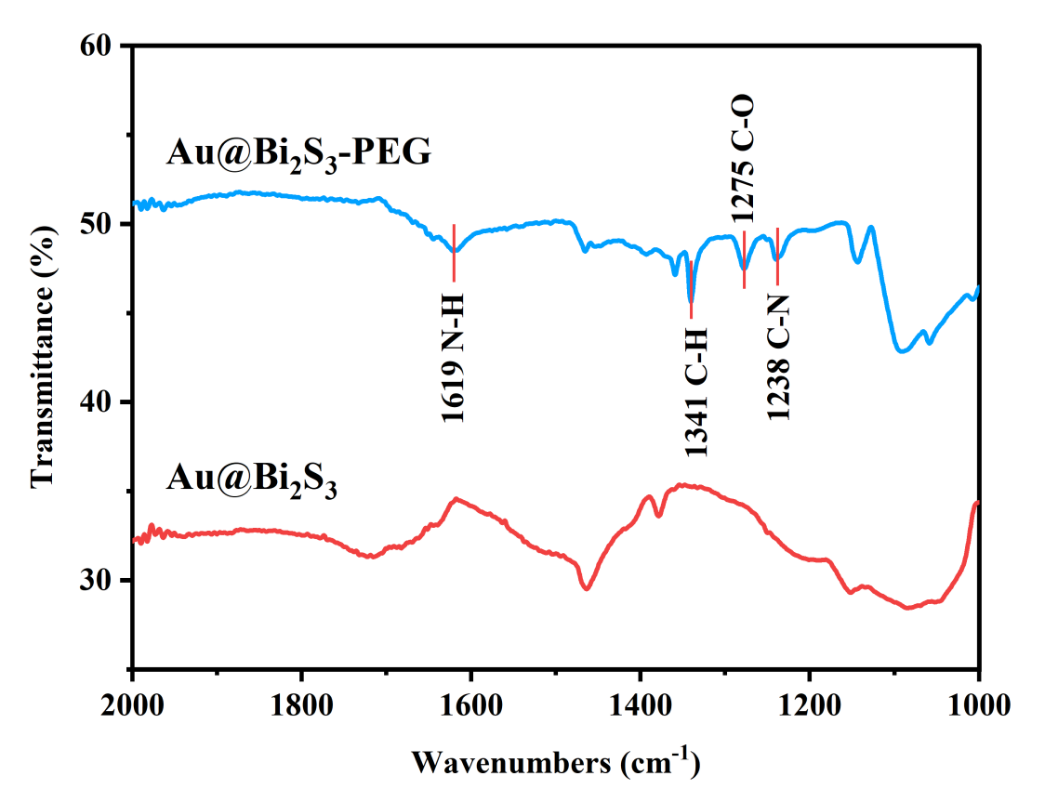


**Figure S9.** Fourier transform infrared spectrum of Au@Bi_2_S_3_ and Au@Bi_2_S_3_-PEG.


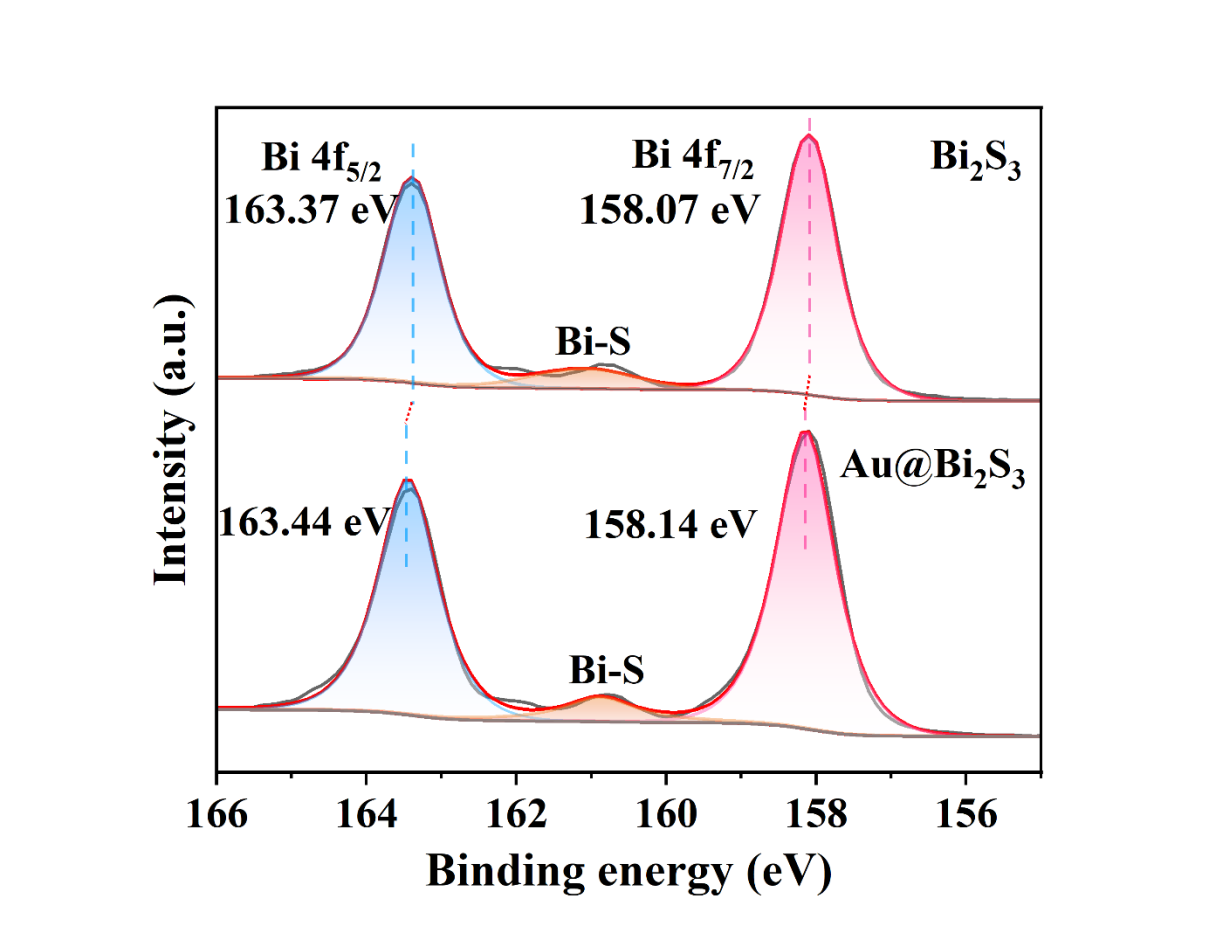


**Figure S10.** XPS high-resolution of Bi 4f for Bi_2_S_3_ and Au@Bi_2_S_3_, respectively.


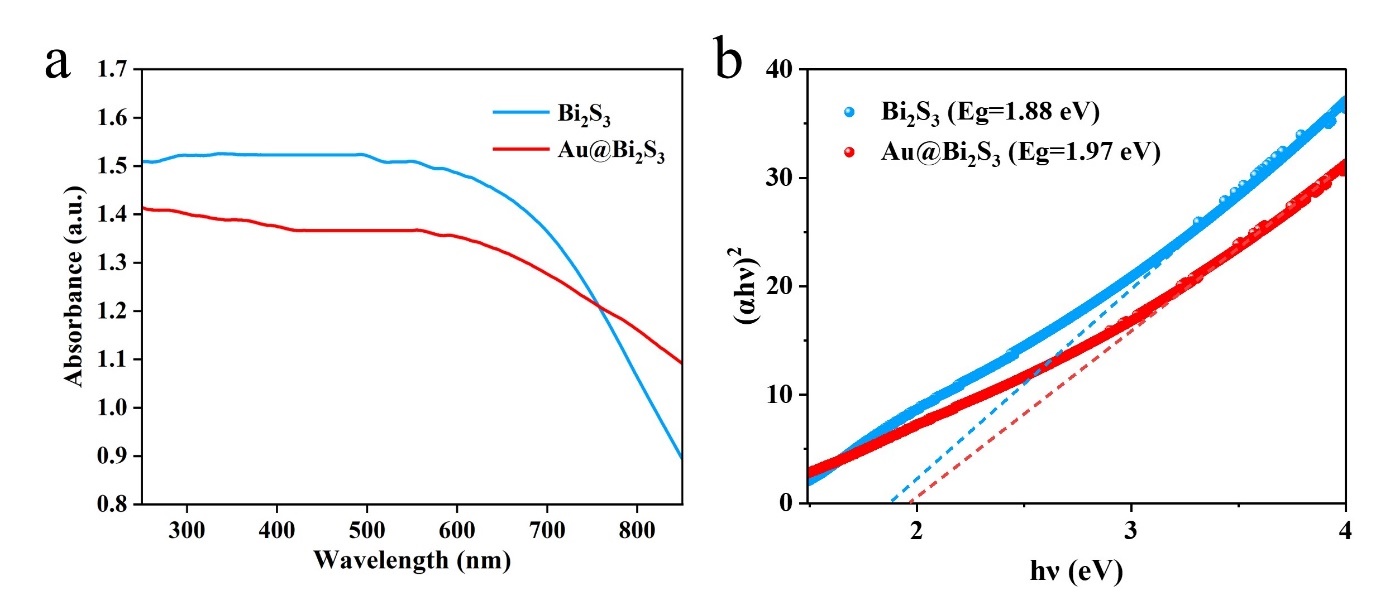


**Figure S11.** a): The UV-Vis spectra and b): Tauc plot of Bi_2_S_3_ and Au@Bi_2_S_3_ nanoreactors, Bi_2_S_3_ and Au@Bi_2_S_3_ have bandgaps of 1.88 eV and 1.97 eV, respectively.


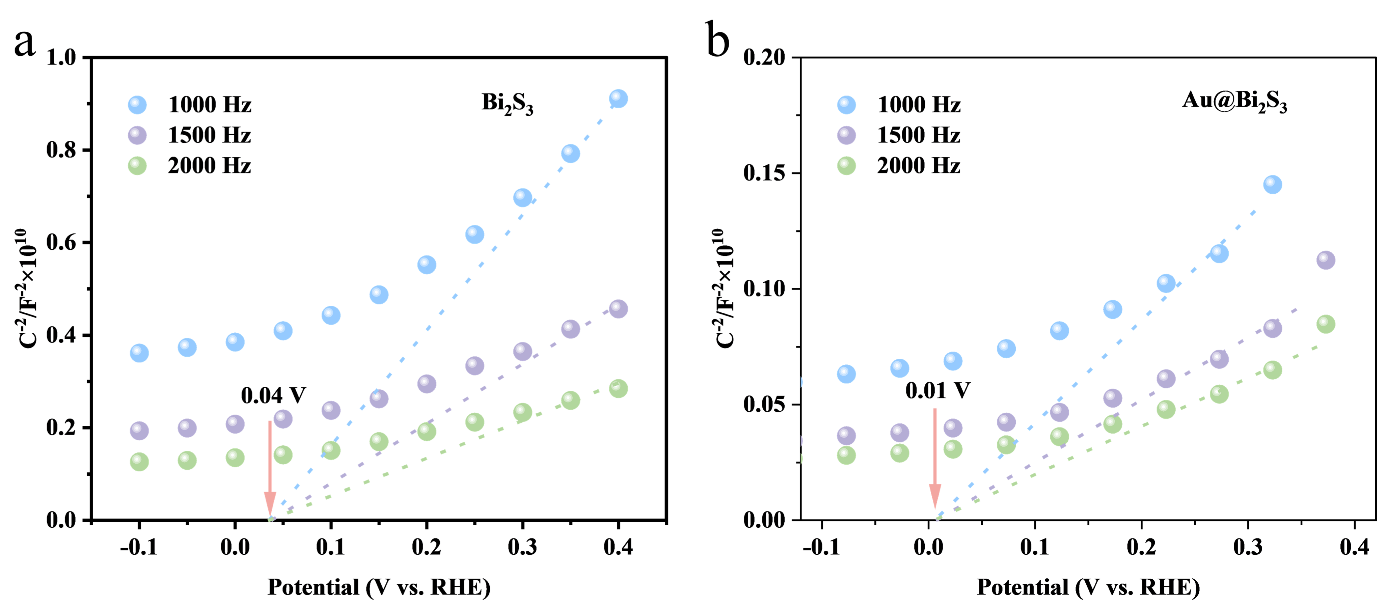


Figure S12. a-b): The Mot-Schottky plots of Bi_2_S_3_ and Au@Bi_2_S_3_ at different frequencies.


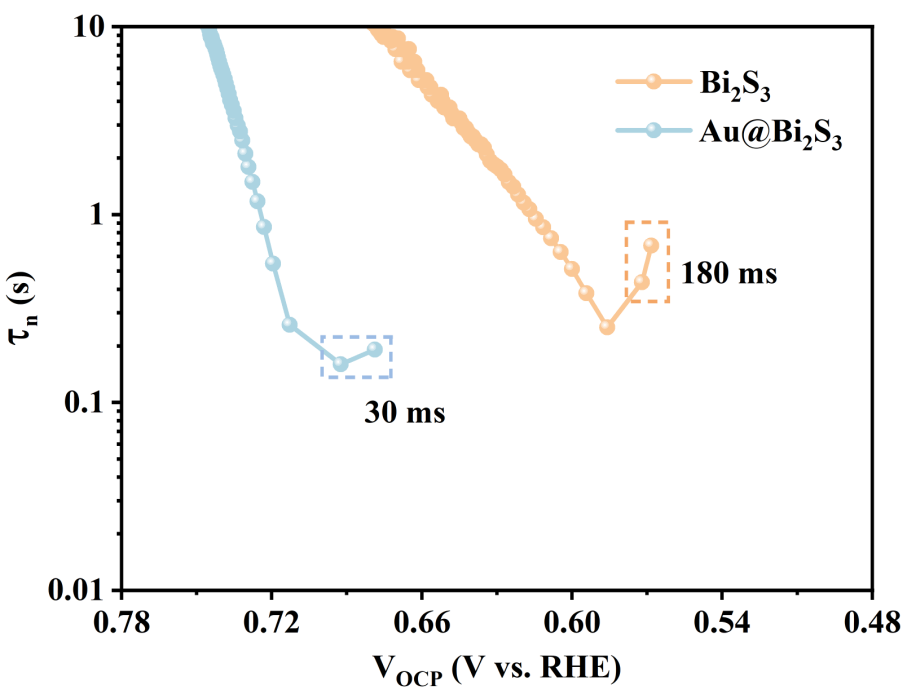


Figure S13. Carrier lifetime derived from OCP decay curve of Bi_2_S_3_ and Au@Bi_2_S_3_.


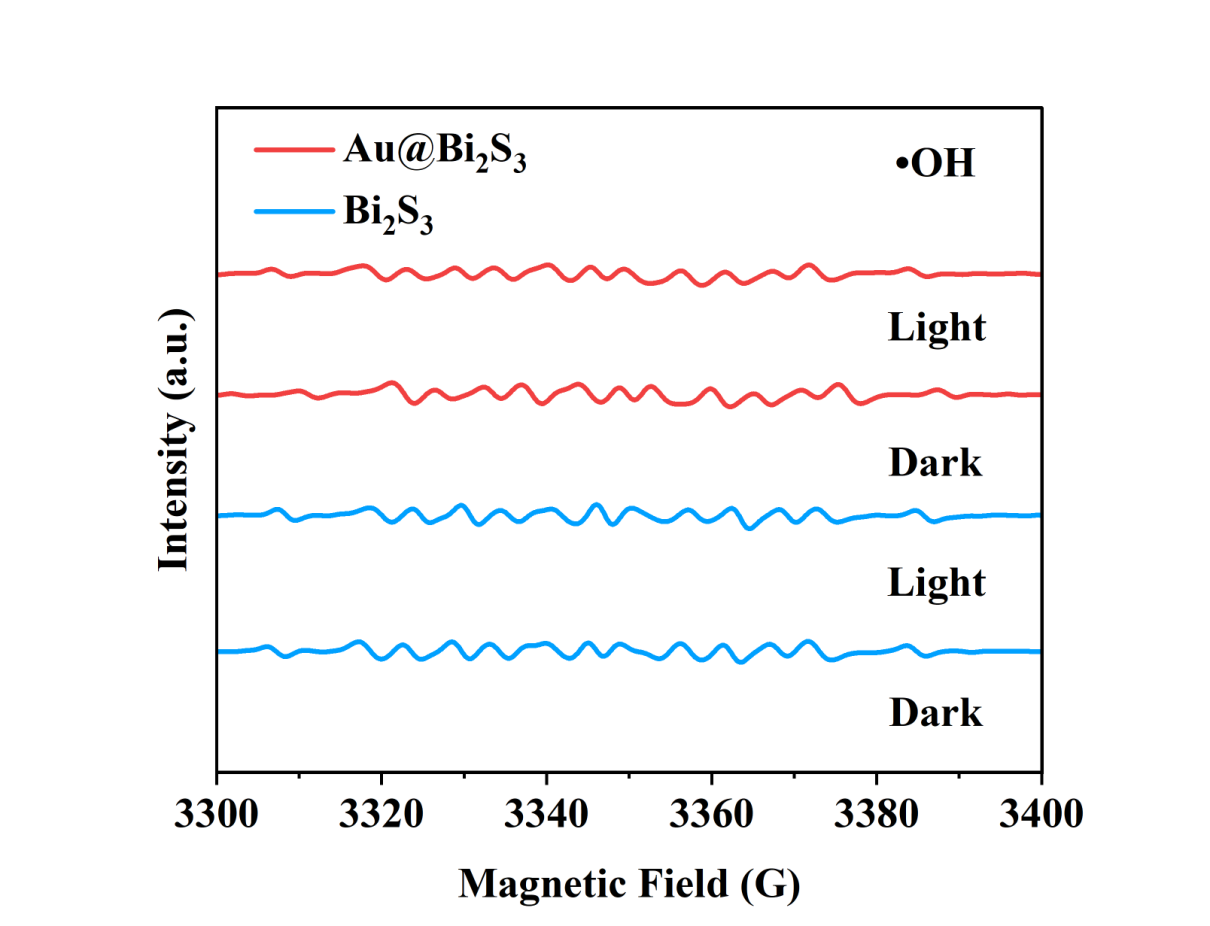


Figure S14. EPR spectra of Bi_2_S_3_ and Au@Bi_2_S_3_ for measuring •OH.


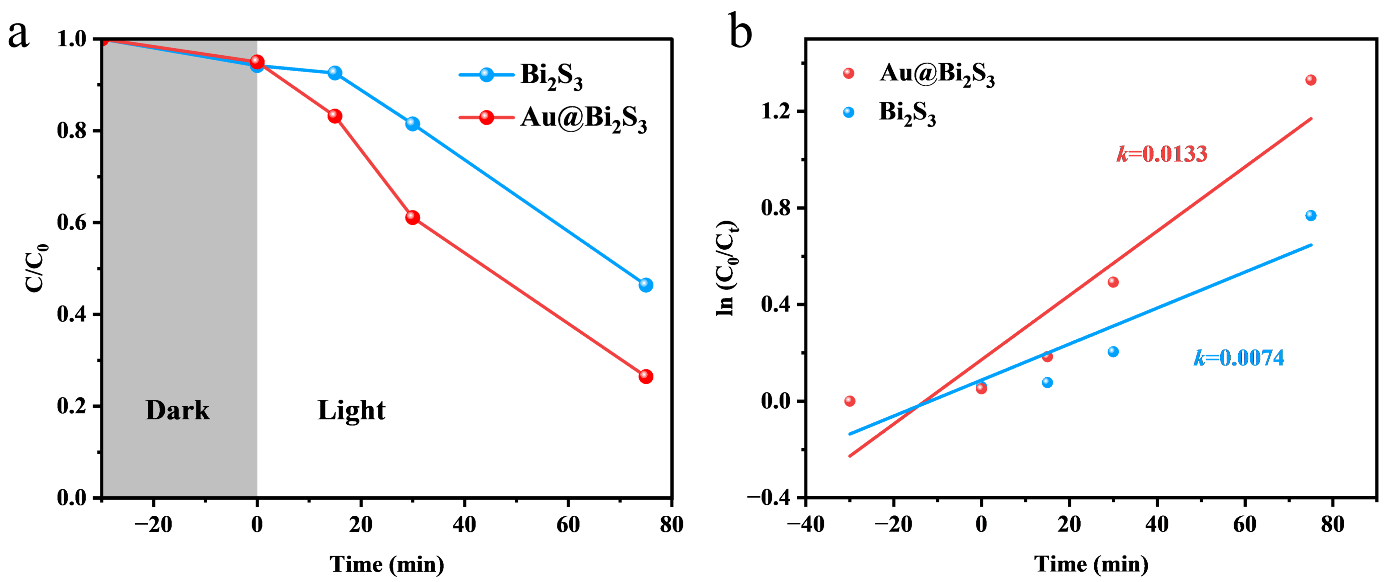


Figure S15. a): Photocatalytic degradation of methylene blue by Bi_2_S_3_ and Au@Bi_2_S_3_; b): First-order kinetic reaction rate of Bi_2_S_3_ and Au@Bi_2_S_3_.


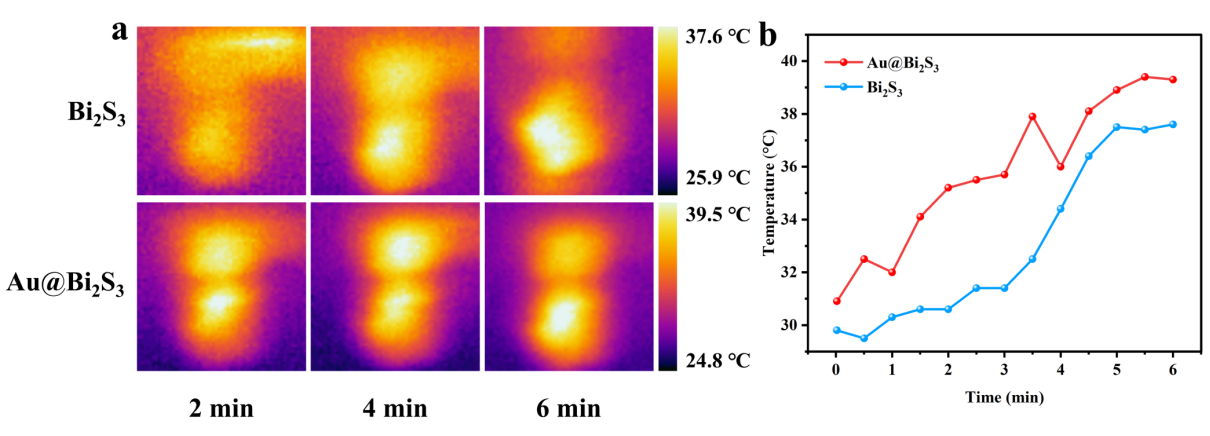


Figure S16. a): Bi_2_S_3_ and Au@Bi_2_S_3_ of infrared thermal image for 2, 4 and 6 minutes; b): Temperature–time plots of Bi_2_S_3_ and Au@Bi_2_S_3_.


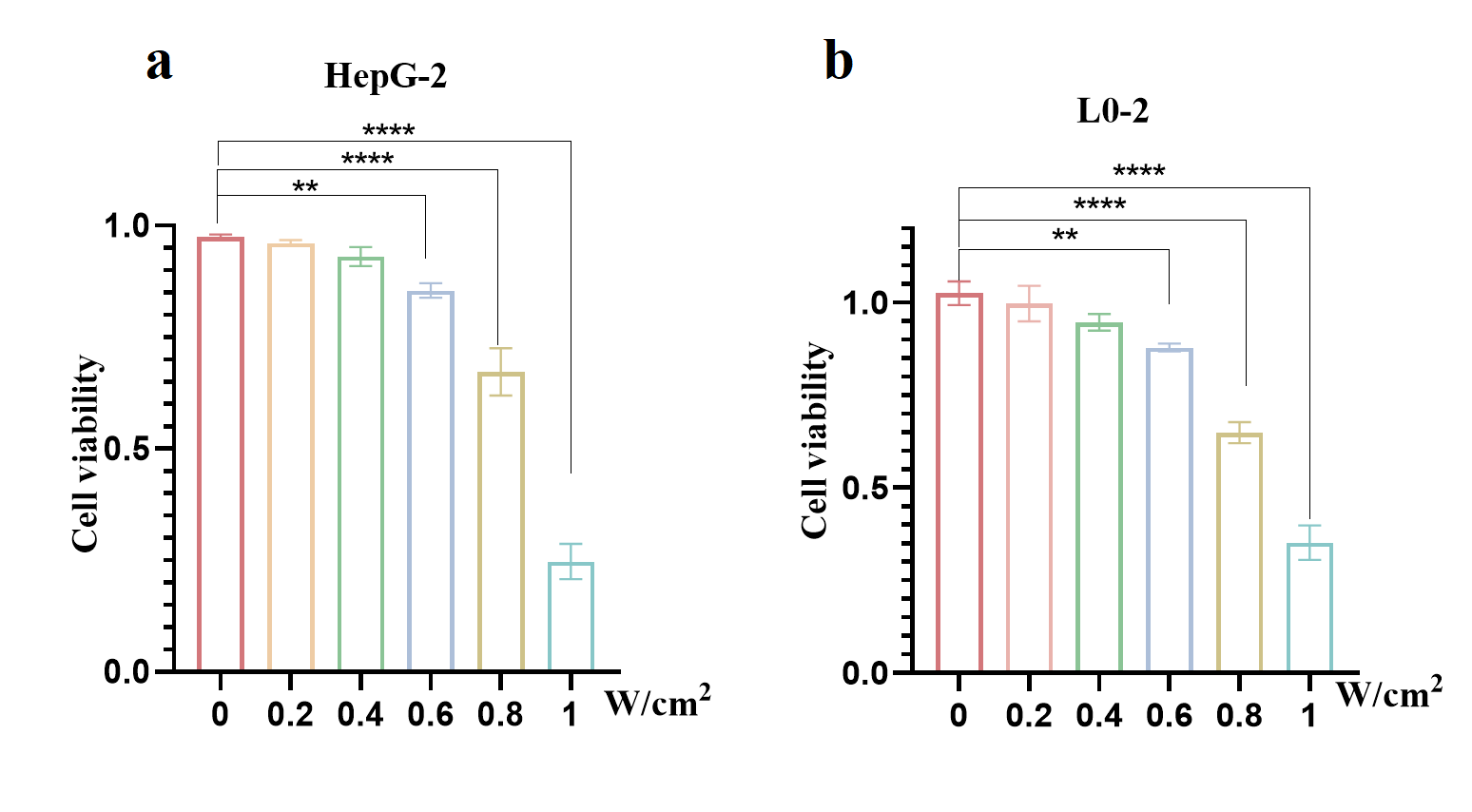


Figure S17. a): CCK-8 was used to detect the toxicity of different light intensities on HepG-2 cells; b): CCK-8 was used to detect the toxicity of different light intensities on L-02 cells. All results of this study were derived from three independent experiments. **p< 0.05, **p< 0.01, ***p< 0.001, ****p< 0.0001*. Error bars represent SEM.


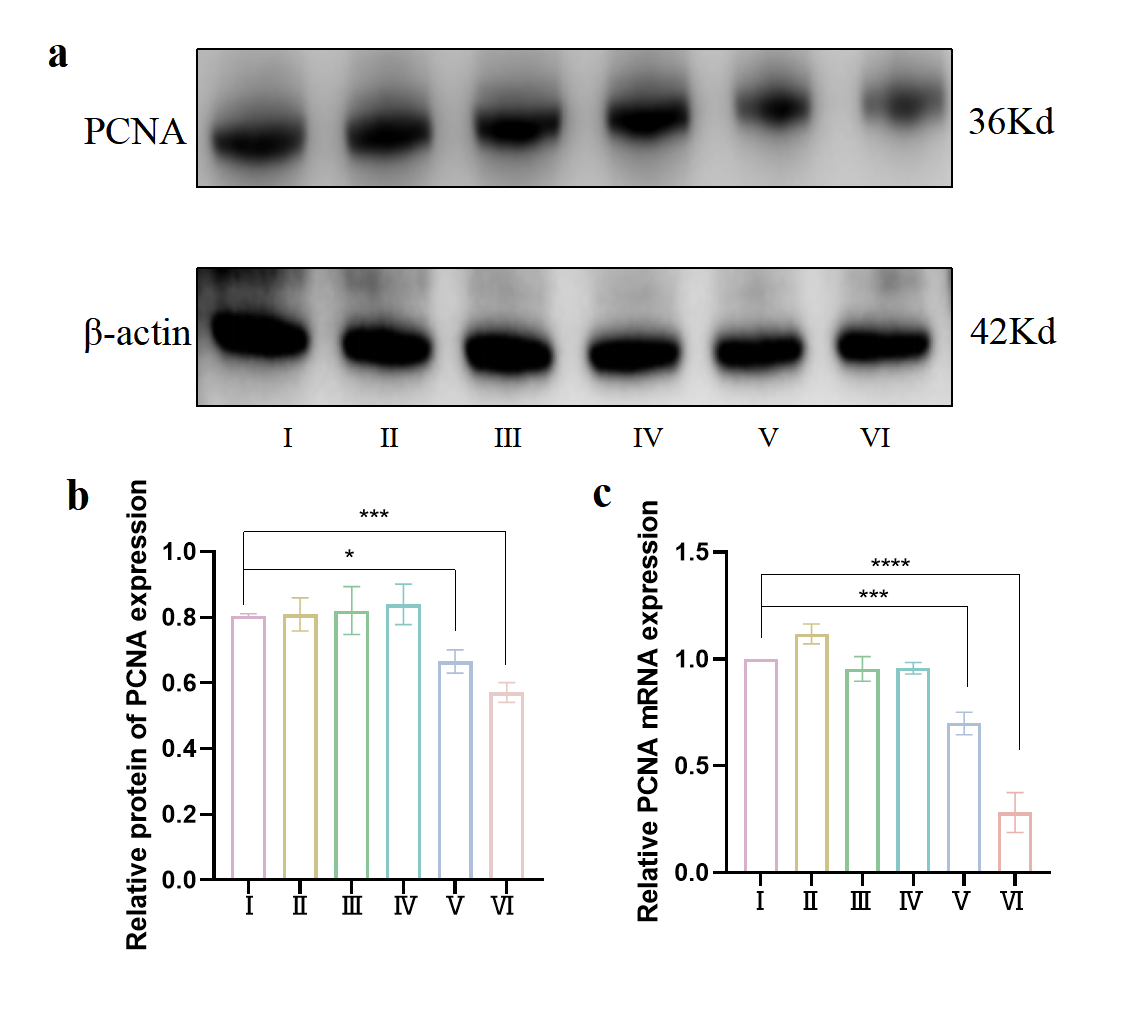


**Figure S18.** a): The expression of proliferation-related protein PCNA was detected by using Western blotting; b): The expression of PCNA protein was semi-quantitatively; c): The expression of PCNA mRNA was detected by RT-qPCR. (I: Control; Ⅱ: Light for 30 minutes; III: Bi_2_S_3_; IV: Au@Bi_2_S_3_; V: Bi_2_S_3_ under light; VI: Au@Bi_2_S_3_ under light.) All results of this study were derived from three independent experiments. **p< 0.05, **p< 0.01, ***p< 0.001, ****p< 0.0001.* Error bars represent SEM.


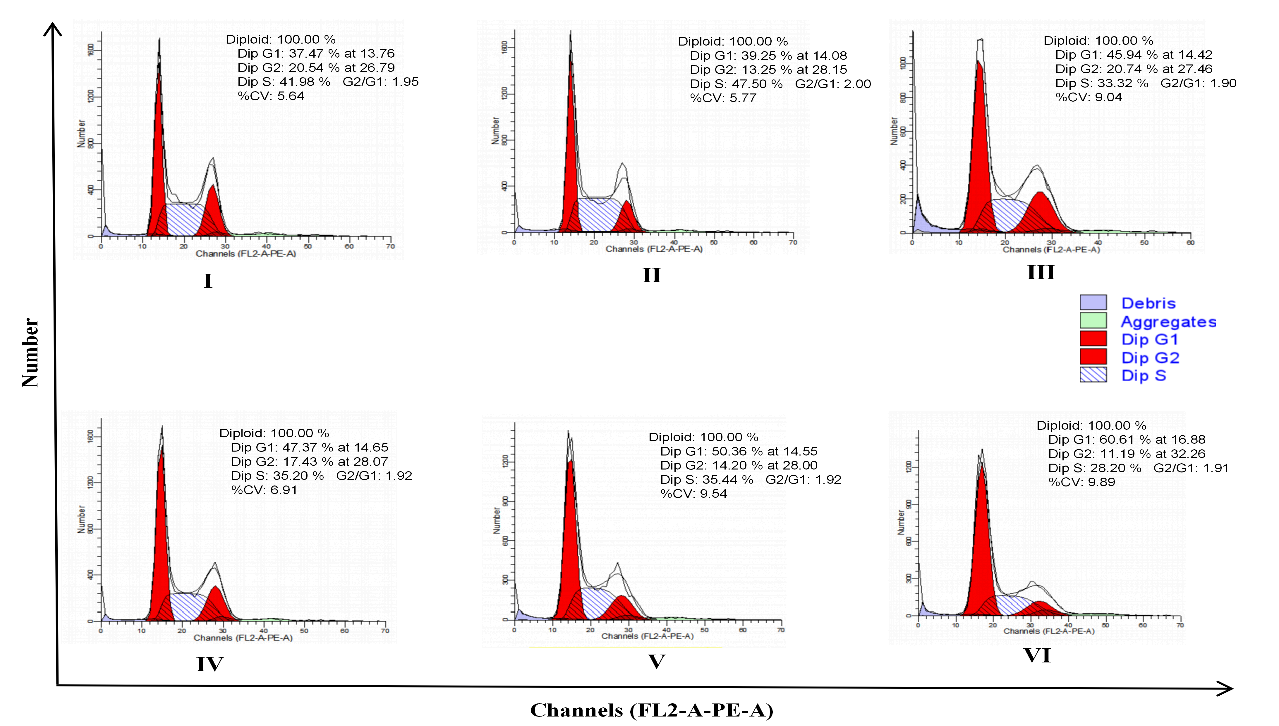


**Figure S19.** Flow cytometry was used to detect cell cycle changes in six groups. (I: Control; Ⅱ: Light for 30 minutes; III: Bi_2_S_3_; IV: Au@Bi_2_S_3_; V: Bi_2_S_3_ under light; VI: Au@Bi_2_S_3_ under light.)


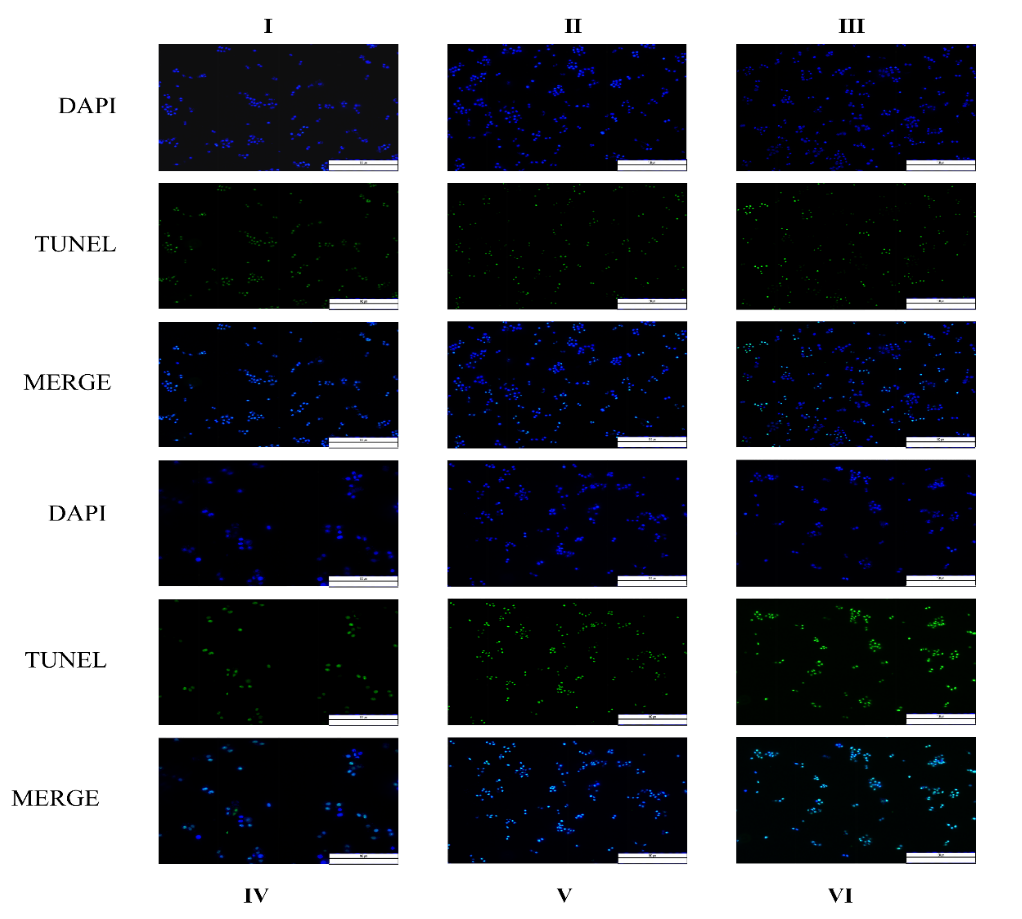


**Figure S20.** TUNEL fluorescence staining was used to detect the effects of different treatments on HepG-2 cell apoptosis. (I: Control; Ⅱ: Light for 30 minutes; III: Bi_2_S_3_; IV: Au@Bi_2_S_3_; V: Bi_2_S_3_ under light; VI: Au@Bi_2_S_3_ under light.)


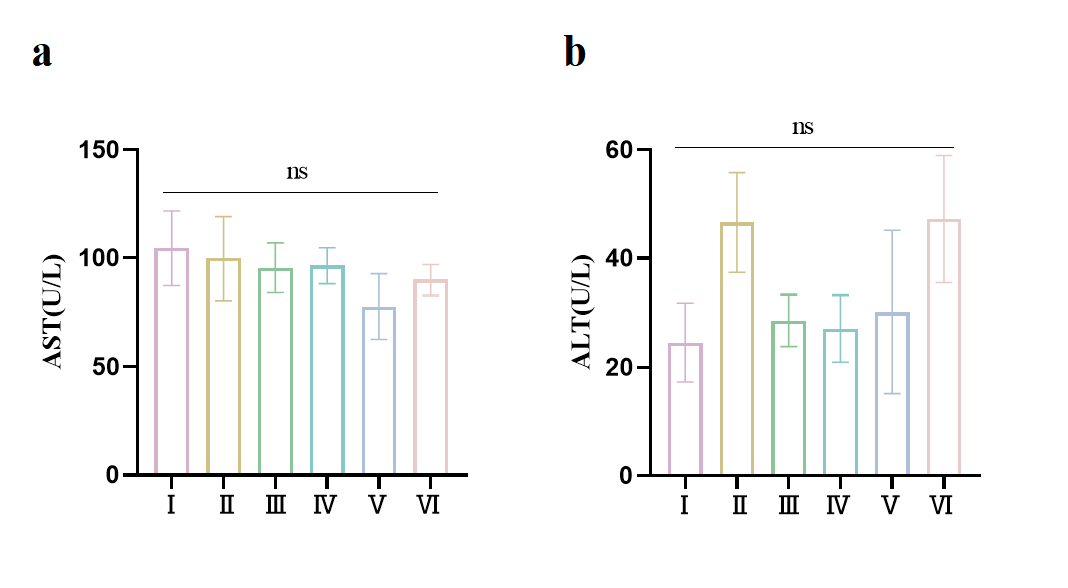


**Figure S21.** a): The AST level was detected in mice after 7-day different treatments; b): The ALT level was detected in mice after 7-day different treatments. (I: Control; Ⅱ: Light for 30 minutes; III: Bi_2_S_3_; IV: Au@Bi_2_S_3_; V: Bi_2_S_3_ under light; VI: Au@Bi_2_S_3_ under light.) All results of this study were derived from three independent experiments. **p< 0.05, **p< 0.01, ***p< 0.001, ****p< 0.0001.* Error bars represent SEM.


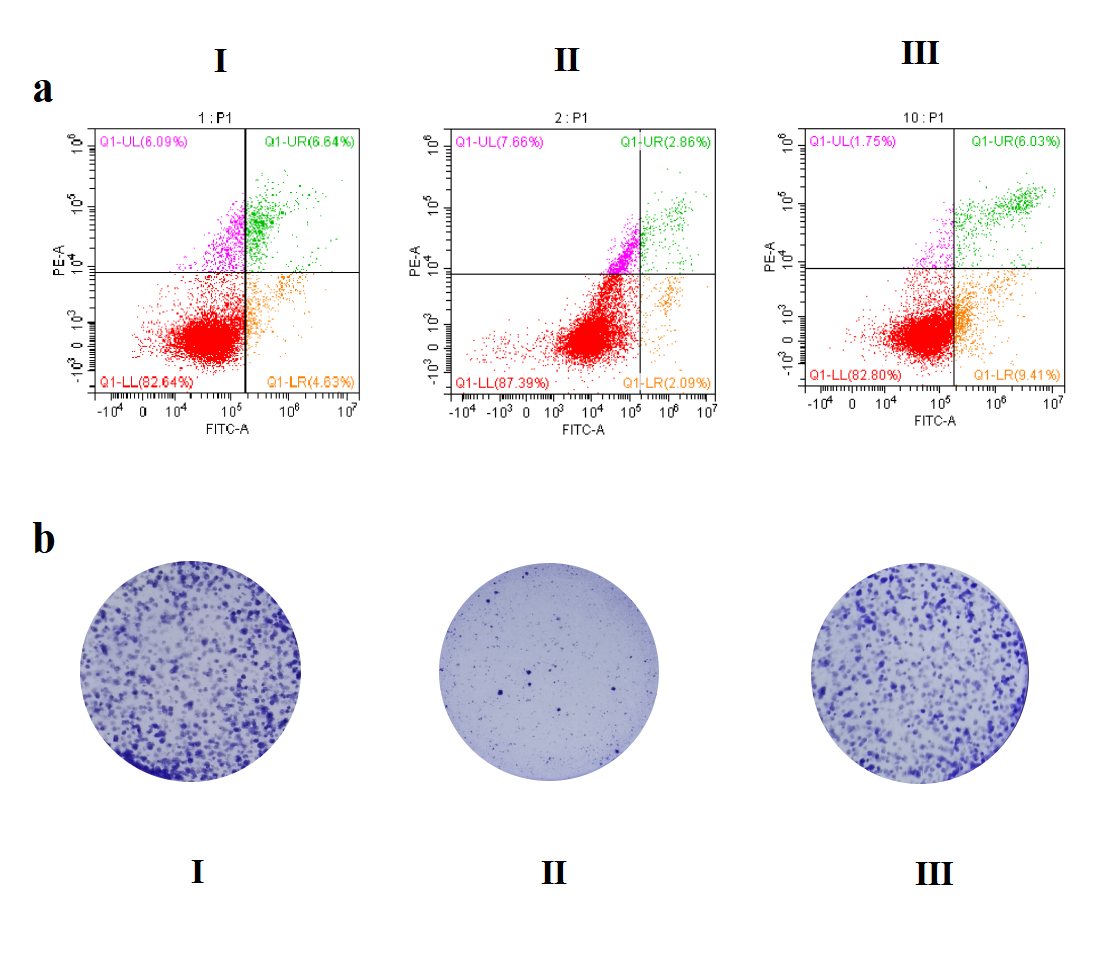


**Figure S22.** a): Flow cytometry was used to detect changes in cell apoptosis in the three groups; b): The clonal formation experiment was used to detect changes in cell proliferation in the three groups. (Ⅰ: control; Ⅱ: Au@Bi_2_S_3_ under light; Ⅲ: Au@Bi_2_S_3_ under light+XMU-MP-1.)


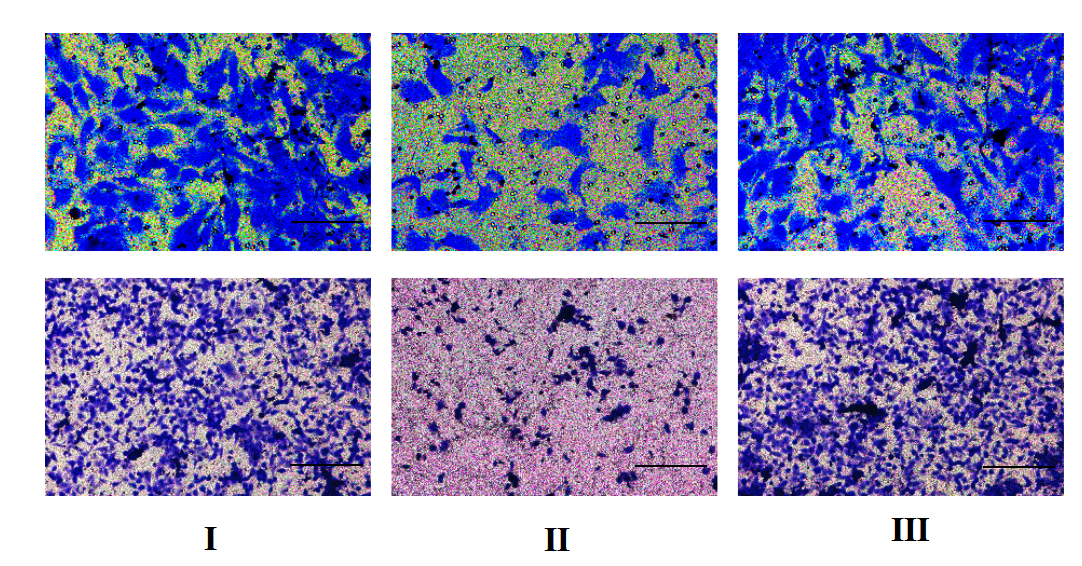


**Figure S23.** Tranwell was used to detect changes in cell migration and invasion in the three groups. (Ⅰ: control; Ⅱ: Au@Bi_2_S_3_ under light; Ⅲ: Au@Bi_2_S_3_ under light+XMU-MP-1.)


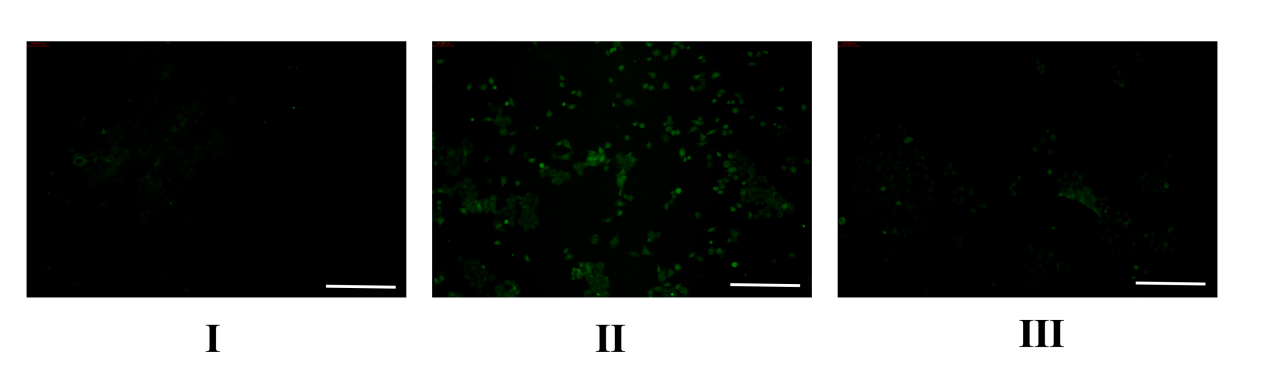


**Figure S24.** The DCFH-DA probe was used to detect changes in ROS in three groups. (Ⅰ: control; Ⅱ: Au@Bi_2_S_3_ under light; Ⅲ: Au@Bi_2_S_3_ under light+XMU-MP-1.)


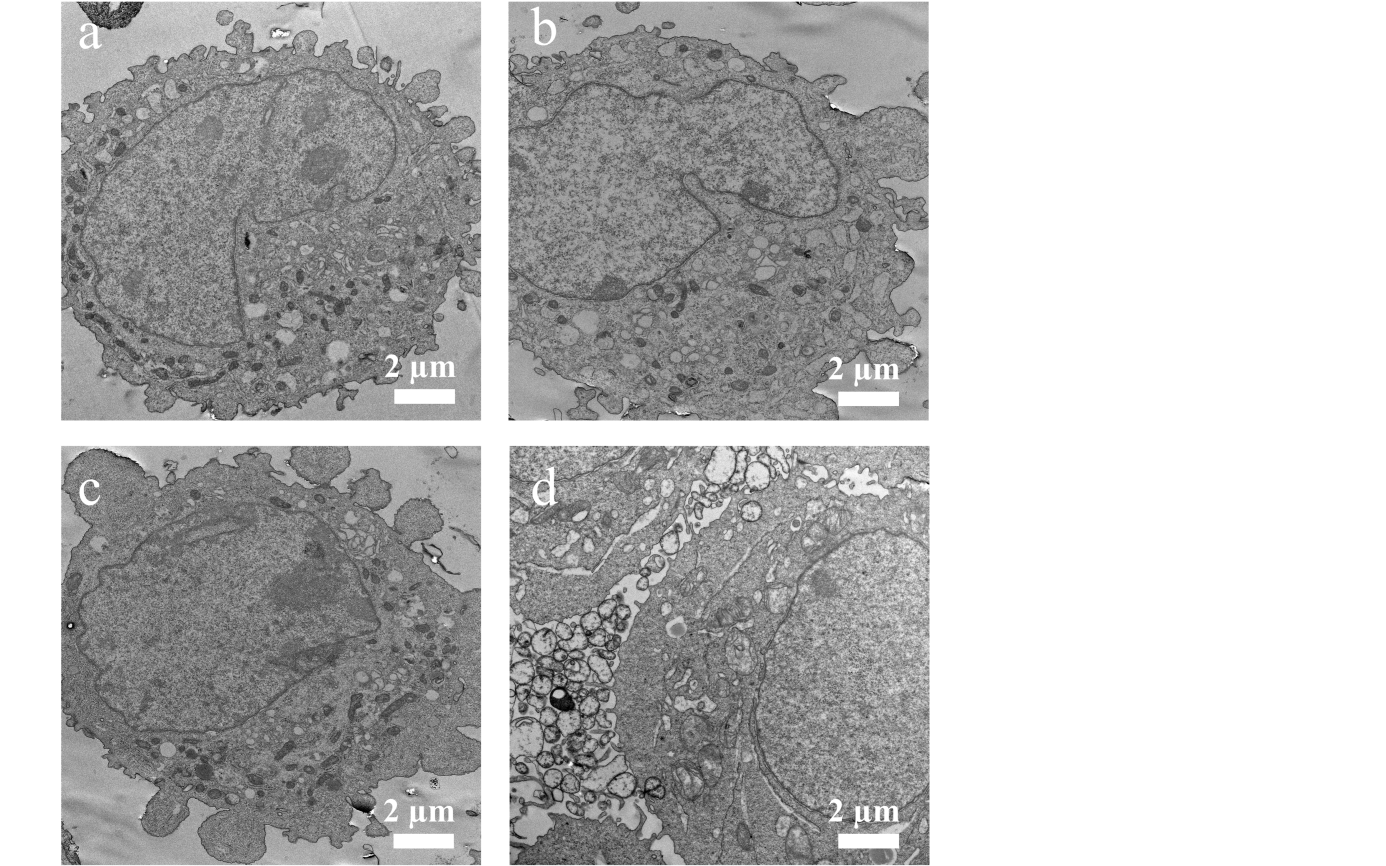


**Figure S25.** The TEM of HepG-2 cells was been observed under different treatment conditions. a): Untreated control cells; b): Cells subjected to light irradiation for 30 minutes; c): Cells stimulated with 64 μg/mL Au@Bi_2_S_3_ nanoreactors; d): Cells co-treated with 64 μg/mL Au@Bi_2_S_3_ nanoreactors followed by 30 minutes light irradiation.


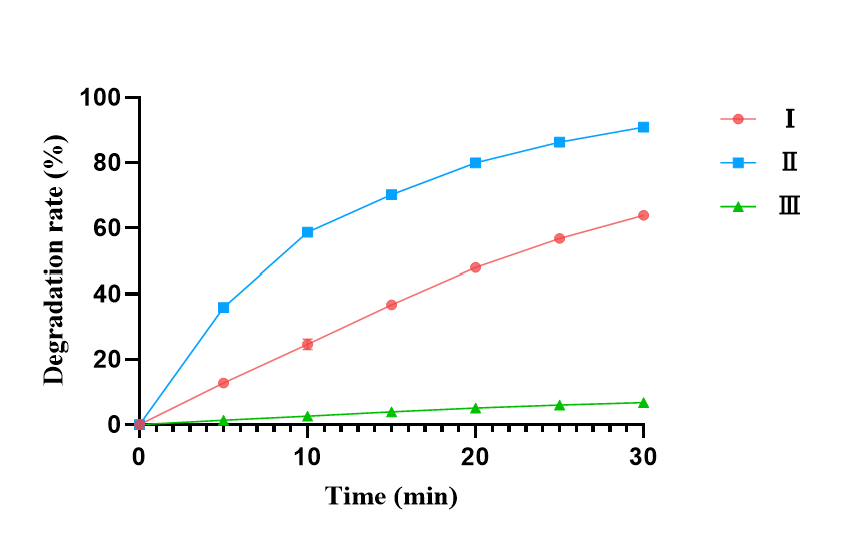


**Figure S26.** Kinetic curve of ABDA degradation was calculated. Ⅰ: Au@Bi_2_S_3_; Ⅱ: Bi_2_S_3_; Ⅲ: control.


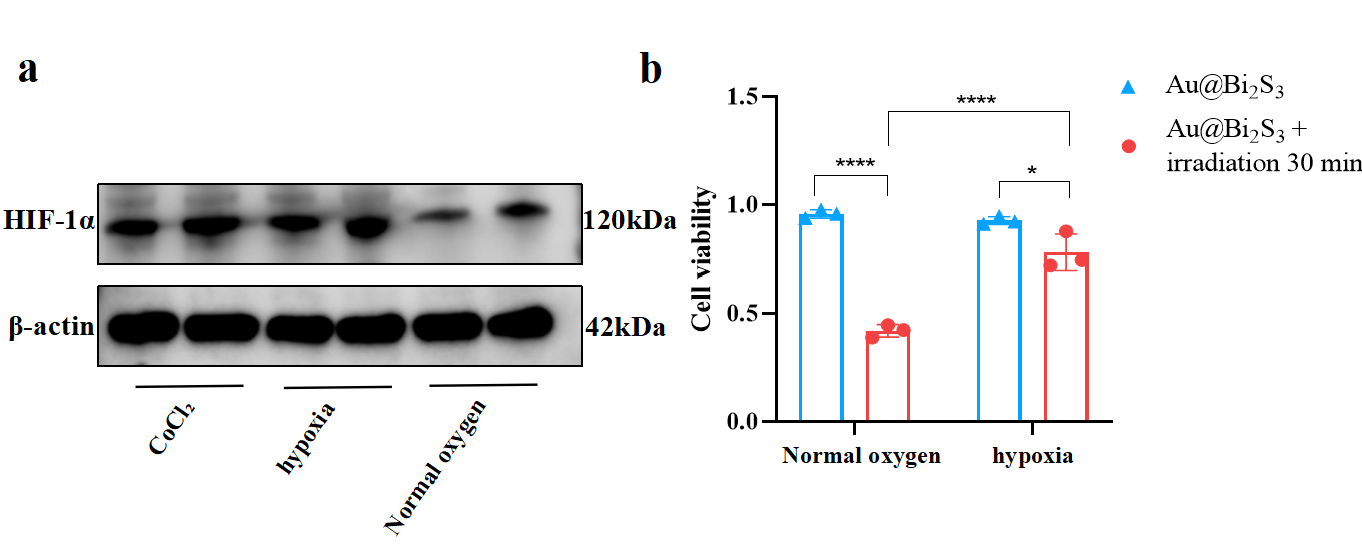


Figure S27. a): Western blotting was used to detect the expression of hypoxia index protein HIF-1α in HepG-2 cells in normal oxygen group (20% O_2_), hypoxia group (1% O_2_) and CoCl_2_ treatment group; b): CCK-8 was used to detect the effects of Au@Bi_2_S_3_ and Bi_2_S_3_ on the cell viability of HepG-2 cells under hypoxia and normal oxygen, respectively. **p< 0.05, **p< 0.01, ***p< 0.001, ****p< 0.0001.* Error bars represent SEM.


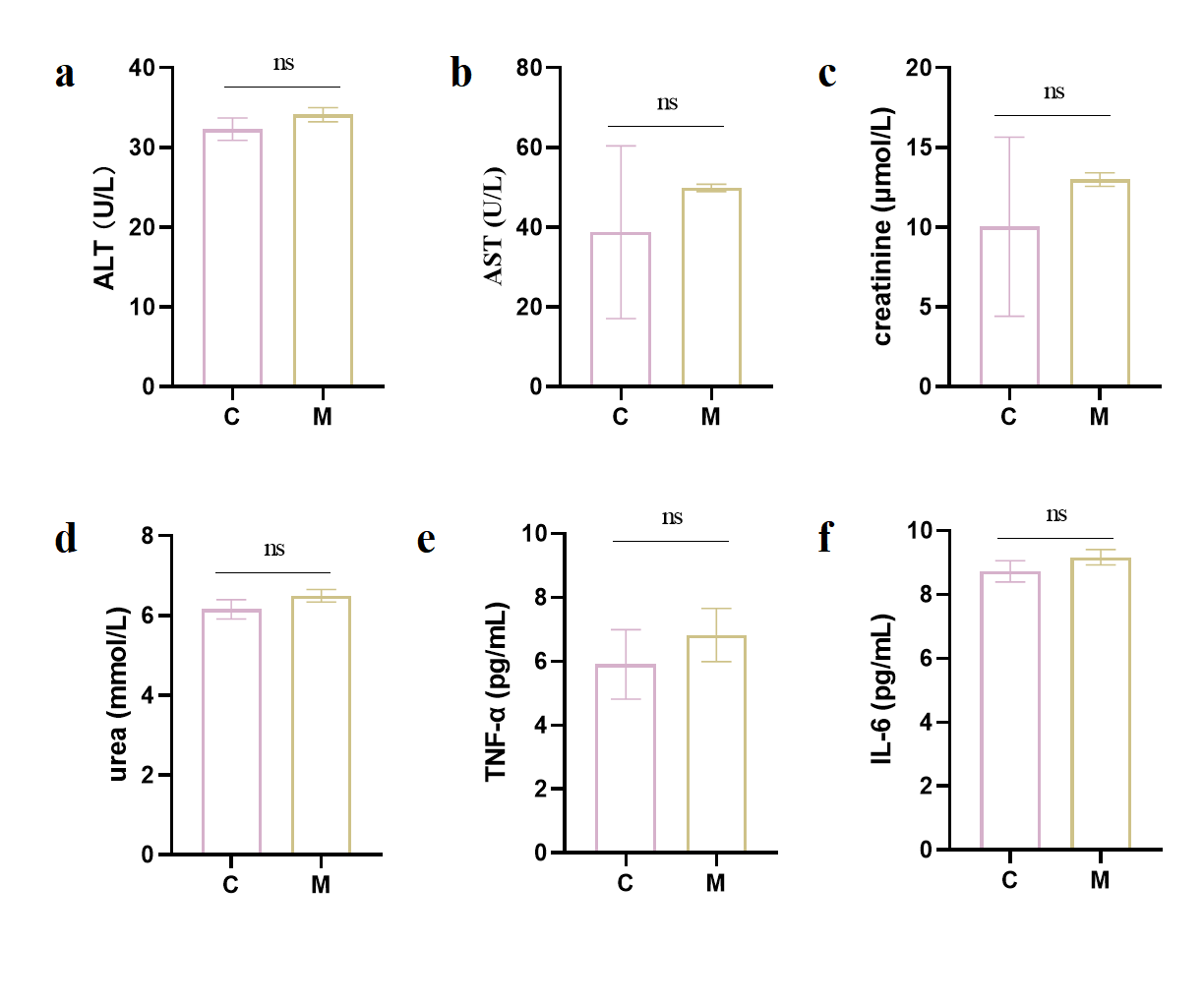


**Figure S28.** a-d): ALT, AST, creatinine and urea levels were detected in mice after 14-day different treatments; e, f): ELISA was used to detect the expression changes of inflammatory cytokines TNF-α and IL-6 in serum. C: normal control; M: treated with Au@Bi_2_S_3_. Error bars represent SEM.


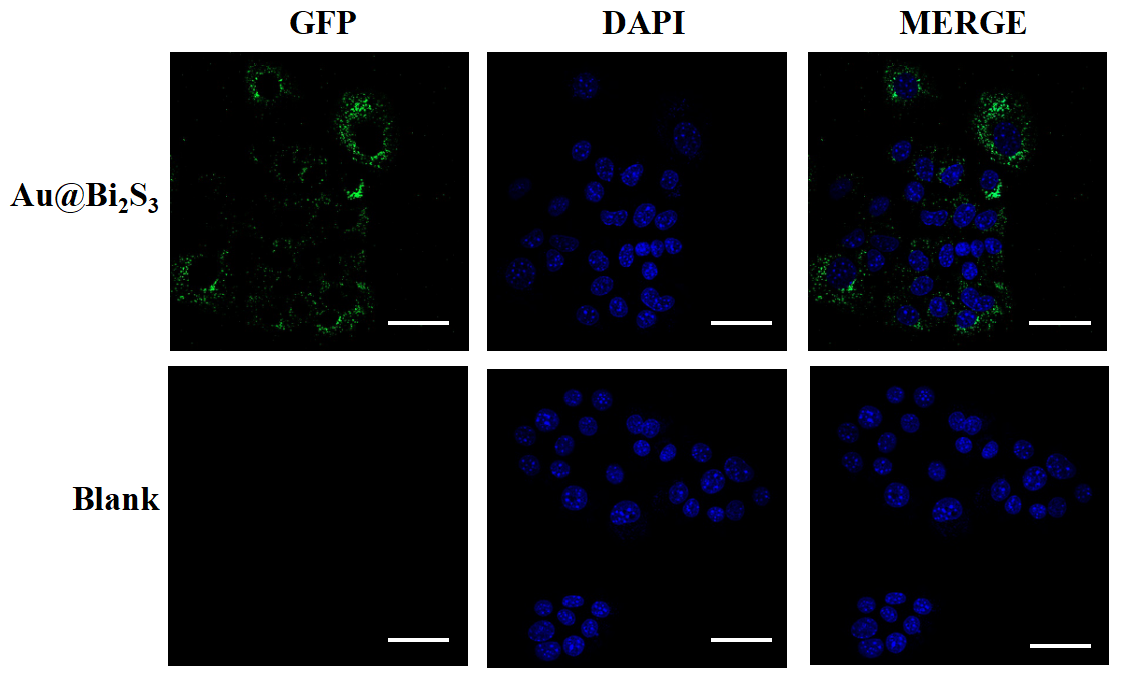


**Figure S29.** The uptake of Au@Bi_2_S_3_ by HepG-2 cells was detected by immunofluorescence.


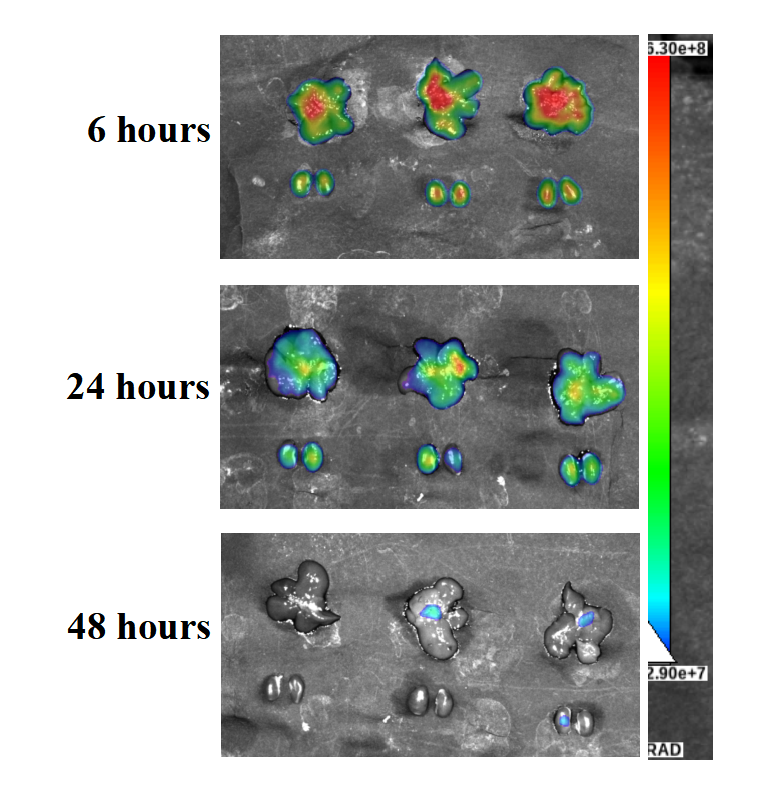


**Figure S30.** Optical in vivo imaging was used to detect the accumulation of Au@Bi_2_S_3_ in liver and kidney of mice after 6 hours, 24 hours and 48 hours.

**Table 2.** Comparison of photodynamic therapy of HCC.

| **Photocatalyst** | **Irradiation wavelength (nm)** | **Tumor suppression rate (%)** | **Time (d)** | **Ref.** |
| --- | --- | --- | --- | --- |
| **Au@Bi_2_S_3_** | 640-710 | 93 | 20 | This work |
| **TB/PTX@RTK** | 540 | 91.7 | 21 | [1] |
| **BLICP@O_2_** | 1064 | 90.4 | 18 | [2] |
| **HCPT@NMOFs-RGD** | 655 | 88.6 | 12 | [3] |
| **ZnPc** | 400-700 | >80 | 4 | [4] |
| **ICG-lactosome** | 570 | 66.9 | 8 | [5] |
| **NM-HB NPs** | 488/635 | 63.6 | 28 | [6] |
| **PS-GNPs** | 640-710 | 18.6 | 28 | [7] |

**References**

1. Xu, J., Zheng, Q., Cheng, X., Hu, S., Zhang, C., Zhou, X., Sun, P., Wang, W., Su, Z., Zou, T., Song, Z., Xia, Y., Yi, X., & Gao, Y. Chemo-photodynamic Therapy with Light-triggered Disassembly of Theranostic Nanoplatform in Combination with Checkpoint Blockade for Immunotherapy of Hepatocellular carcinoma. J. Nanobiotechnology. 2021, 19(1), 355. <https://doi.org/10.1186/s12951-021-01101-1>
2. Zeng, S., Chen, J., Gao, R., Chen, R., Xue, Q., Ren, Y., Liu, L., Tang, C., Hu, H., Zeng, N., Wen, S., Zhang, H., Liu, C., & Fang, C. NIR-II Photoacoustic Imaging-Guided Oxygen Delivery and Controlled Release Improves Photodynamic Therapy for Hepatocellular Carcinoma. Adv. Mater. 2024, 36(4), e2308780. <https://doi.org/10.1002/adma.202308780>
3. Shang, Y., Zhang, H., Cheng, Y., Cao, P., Cui, J., Yin, X., Fan, S., & Li, Y. Fluorescent Imaging-Guided Chemo- and Photodynamic Therapy of Hepatocellular Carcinoma with HCPT@NMOFs-RGD Nanocomposites. Int J Nanomedicine. 2022, 17, 1381–1395. <https://doi.org/10.2147/IJN.S353803>
4. Ogbodu, R. O., Nitzsche, B., Ma, A., Atilla, D., Gürek, A. G., & Höpfner, M. Photodynamic Therapy of Hepatocellular Carcinoma Using Tetra-triethyleneoxysulfonyl Zinc Phthalocyanine as Photosensitizer. J Photoch Photobio B. 2020, 208, 111915. <https://doi.org/10.1016/j.jphotobiol.2020.111915>
5. Tsuda, T., Kaibori, M., Hishikawa, H., Nakatake, R., Okumura, T., Ozeki, E., Hara, I., Morimoto, Y., Yoshii, K., & Kon, M. Near-infrared Fluorescence Imaging and Photodynamic Therapy with Indocyanine Green Lactosome has Antineoplastic Effects for Hepatocellular Carcinoma. PloS one, 2017, 12(8), e0183527. <https://doi.org/10.1371/journal.pone.0183527>
6. Zhang, Z., Li, D., Cao, Y., Wang, Y., Wang, F., Zhang, F., & Zheng, S. Biodegradable Hypocrellin B Nanoparticles Coated with Neutrophil Membranes for Hepatocellular Carcinoma Photodynamics Therapy Effectively via JUNB/ROS Signaling. Int Immunopharmacol. 2021, 99, 107624. <https://doi.org/10.1016/j.intimp.2021.107624>
7. Kwon, J. G., Song, I. S., Kim, M. S., Lee, B. H., Kim, J. H., Yoon, I., Shim, Y. K., Kim, N., Han, J., & Youm, J. B. Pu-18-N-butylimide-NMGA-GNP Conjugate is Effective Against Hepatocellular Carcinoma. IMR. 2013, 2(3), 106–111. <https://doi.org/10.1016/j.imr.2013.05.001>
